# Fluorescent Amino Acid Initiated *de novo* Cyclic Peptides for the Label‐Free Assessment of Cell Permeability**

**DOI:** 10.1002/cmdc.202100315

**Published:** 2021-07-28

**Authors:** Yuteng Wu, M. Teresa Bertran, James Rowley, Ewen D. D. Calder, Dhira Joshi, Louise J. Walport

**Affiliations:** ^1^ Protein-Protein Interaction Laboratory The Francis Crick Institute London NW1 1AT UK; ^2^ Department of Chemistry Molecular Sciences Research Hub Imperial College London London W12 0BZ UK; ^3^ Peptide Chemistry The Francis Crick Institute London NW1 1AT UK

**Keywords:** RaPID, peptides, label-free, cell permeability, fluorescence imaging

## Abstract

The major obstacle in applying peptides to intracellular targets is their low inherent cell permeability. Standard approaches to attach a fluorophore (e. g. FITC, TAMRA) can change the physicochemical properties of the parent peptide and influence their ability to penetrate and localize in cells. We report a label‐free strategy for evaluating the cell permeability of cyclic peptide leads. Fluorescent tryptophan analogues 4‐cyanotryptophan (4CNW) and β‐(1‐azulenyl)‐L‐alanine (AzAla) were incorporated into *in vitro* translated macrocyclic peptides by initiator reprogramming. We then demonstrate these efficient blue fluorescent emitters are good tools for monitoring peptide penetration into cells.

## Graphical abstract

**Without a trace**: Fluorescent amino acids ClAc−4CNW, ClAc−AzAla were incorporated into *in vitro* translated macrocyclic peptides by genetic code reprogramming. The minimally perturbing dyes were found to be good tools for tracking peptide penetration into cells by fluorescence imaging and flow cytometry.

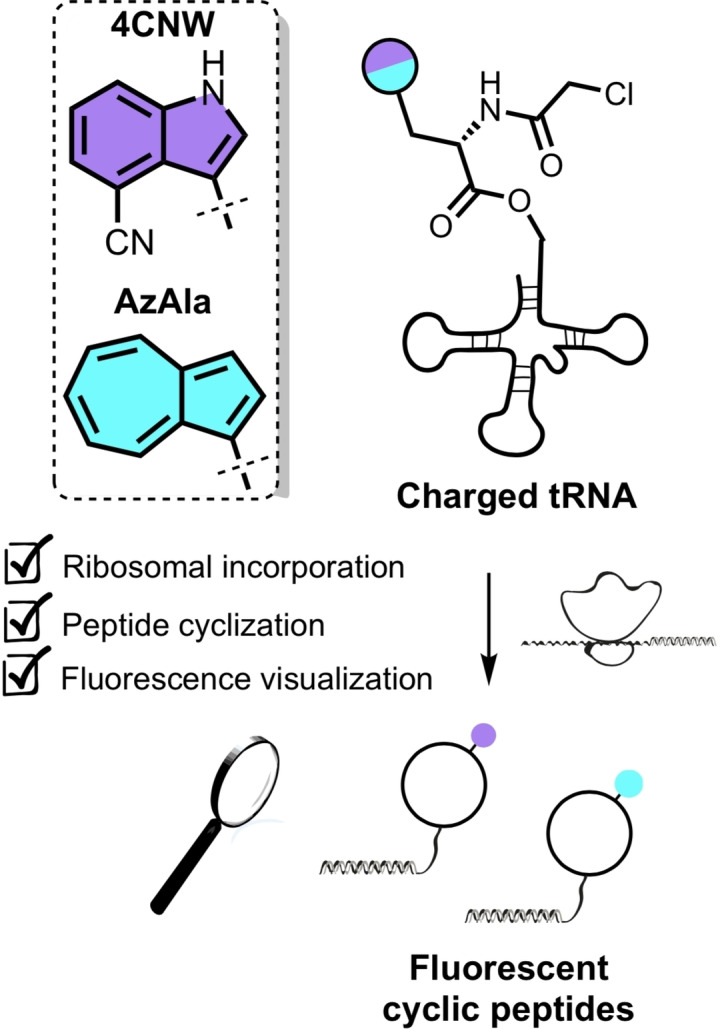

In recent years cyclic peptides have emerged as promising drug candidates that can combine therapeutic benefits from both small molecules and biologics.[^1^, ^2^] They show excellent selectivity and tight binding to their targets, and their ability to interact with shallow surfaces makes them suitable for tackling challenging surfaces such as those involved in protein‐protein interactions.^[3]^ High affinity *de novo* cyclic peptides can be efficiently identified from enormous encoded peptide libraries using e. g. Phage or mRNA display platforms (10^8^–10^14^ members). In particular the Random nonstandard Peptides Integrated Discovery (RaPID) system allows the identification of cyclic peptides containing a wide range of unnatural amino acids.^[4]^

One major challenge for the application of peptides to intracellular targets, however, is their low inherent cell permeability. Straightforward assays are therefore required to assess the permeability of promising leads, with fluorescence imaging being a popular approach. Typically, peptide leads are appended with a fluorophore (e. g. FITC, TAMRA, Rhodamine) to assess cell permeability.^[5]^ Whilst widely used, these strategies use the labelled peptide as a proxy for the untagged version of the molecule. This can result in misleading findings where the fluorophore changes the physicochemical properties of the parent peptide and influences their ability to penetrate and localize in cells.[^6^, ^7^] Use of minimally perturbing dyes or label‐free assays are therefore the gold standard for determining the cell permeability of cyclic peptide leads.

Recently, non‐canonical amino acids 4‐cyanotryptophan (4CNW) and β‐(1‐azulenyl)‐l‐alanine (AzAla) have been shown to be efficient blue fluorescent emitters.[^8^, ^9^] Benefiting from their close structural similarity with tryptophan, incorporation of such molecules into cyclic peptides is potentially less disruptive to overall structure and permeability properties. Inspired by this, we set out to explore the introduction of 4CNW and AzAla into cyclic peptides by ribosomal translation. We selected the flexible *in vitro* translation system (FIT), which uses Flexizymes (small flexible tRNA acylation ribozymes) to prepare non‐proteinogenic acyl‐tRNAs using chemically activated amino acids.[^10^, ^11^]

In a typical RaPID selection, the initiator is reprogrammed with an *N*‐chloroacetylated amino acid that readily undergoes peptide cyclisation with a downstream cysteine after *in vitro* translation of the linear peptide.[^11^, ^12^, ^13^] We designed compounds ClAc−4CNW−CME **7** and ClAc−AzAla−CME **8** to explore the inclusion of fluorescent amino acids into *in vitro* translated cyclic peptides *via* initiator reprogramming. The chloroacetyl (ClAc) group facilitates peptide cyclisation with a cysteine residue following translation, and the cyanomethyl ester (CME) acts as an efficient leaving group for flexizyme‐mediated tRNA charging. Amino acids **3** and **4** were obtained by enzymatically assembling 4‐cyanoindole (**1**) or azulene (**2**) with serine using a tryptophan synthase β‐subunit (TrpB) variant (Tm9D8*) developed by Arnold and co‐workers.^[14]^ Treatment of **3** and **4** with *N*‐(chloroacetoxy)succinimide gave intermediates **5** and **6** in moderate yields. Finally, esterification of the carboxylic acids with chloroacetonitrile installed the CME functionality of **7** and **8** (Scheme 1).

**Scheme 1:**
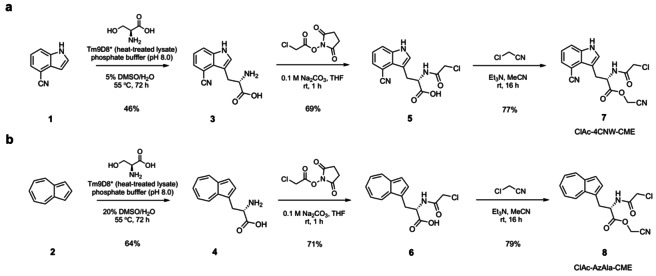
Three‐step preparation of ClAc−4CNW−CME 7 (a) and ClAc−AzAla−CME 8 (b).

With compounds **7** and **8** in hand, we set out to assess their tRNA acylation efficiency by using a tRNA analogue, the microhelix RNA (FAM‐MiHx_23b) (Figure 1). The FAM label allows for visualisation through in gel fluorescence and loading yields were calculated by densitometric analysis of RNA bands. Initial studies were carried out with ClAc−4CNW−CME **7** at pH 7.5. We observed a gradual improvement in acylation yields (33–39 %) as reaction time increased from 2 to 16 h. Altering the pH to 9 significantly improved MiHx charging, reaching 50 % after 2 h, but prolonging incubation time under these conditions did not further increase yields. A similar reactivity profile was observed for ClAc−AzAla−CME **8**, with the higher pH giving the highest MiHx loading (50 %).

**Figure 1:**
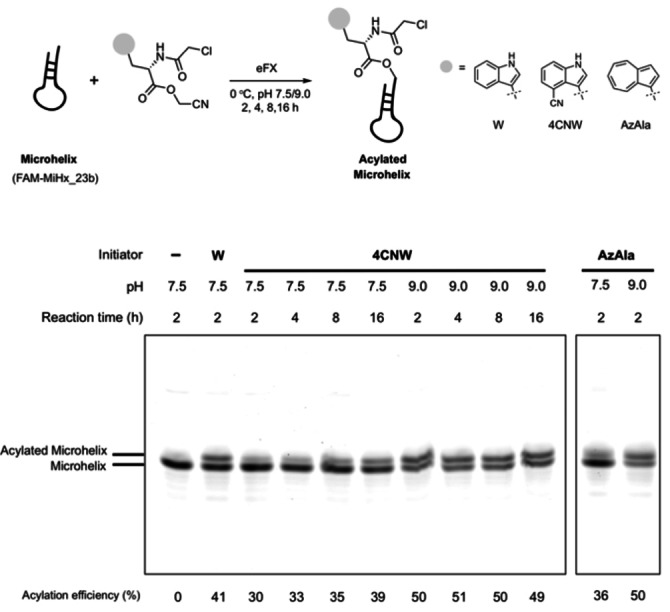
**eFx‐mediated aminoacylation of microhelix RNA (FAM‐MiHx**_**23 b)**. Reaction mixtures were analyzed on a 20 % polyacrylamide gel by detection of the FAM label. Acylation efficiencies were calculated by densitometric analysis of RNA bands. Substrates tested: ClAc−W−CME, ClAc−4CNW−CME **7**, ClAc−AzAla−CME **8**.

Using the optimised conditions (pH 9.0, 2 h) from the MiHx loading assay, substrates **7**, **8** were charged onto initiator tRNA^fMet^_CAU_ through eFx mediated aminoacylation (Figure 2a). The resulting conjugates were then supplemented to a Met‐free FIT system (PURExpress^TM^, Δaa, ΔtRNA, NEB). Synthesis of a model cyclic peptide **P1** initiated with the fluorescent amino acids or ClAc−W as a control, was monitored by MALDI‐TOF mass spectrometry (MS). The translated cyclic peptide products were detected for both fluorescent initiators (Figure 2b). We noted a minor mass peak corresponding to the incorporation of methionine as the initiator was present for all translations (including the control with Cl‐W‐CME), presumably arising from charged tRNA^fMet^ present in the commercial PURExpress kit.

**Figure 2:**
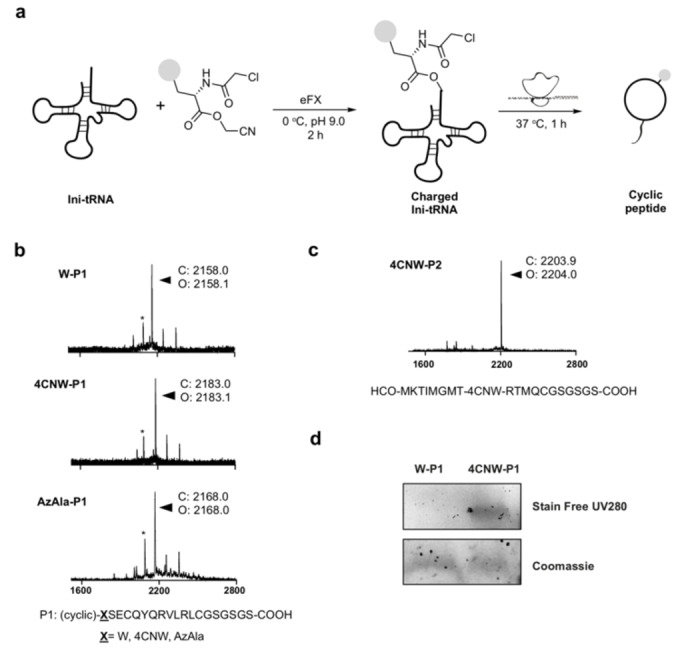
**4CNW/AzAla can be incorporated into a peptide by** ***in vitro*** **translation. a** Charging of initiator tRNA followed by *in vitro* translation to give cyclic peptides. **b** MALDI‐TOF MS spectra of translated cyclic peptides W‐P1, 4CNW‐P1, AzAla‐P1 (*=HCO‐MSECQYQRVLRLCGSGSGS‐COOH). **c** MALDI‐TOF MS spectrum of translated linear peptide 4CNW‐P2. **d** Fluorescence visualization of translated 4CNW‐P1 on a Tricine‐SDS PAGE gel.

We also investigated whether fluorescent amino acids **3**, **4** could be directly incorporated into a model linear sequence **P2** by mischarging onto the tryptophan tRNA by tryptophanyl‐tRNA synthetase. Amino acids **3**, **4** were supplemented to a Trp‐free FIT system. MALDI‐TOF MS analysis showed successful expression of the 4‐cyanotryptophan containing peptide **4CNW**‐**P2**, indicating good tolerance of **3** with tryptophanyl‐tRNA synthetase to allow efficient tRNA charging and subsequent translation (Figure 2c). However, consistent with a previous report,^[15]^ attempts at incorporating AzAla **4** were unsuccessful, presumably due to failed recognition by tryptophanyl‐tRNA synthetase owing to ring expansion and removal of the indole amine.

We then explored the fluorescent properties of the *in vitro* translated peptide containing 4CNW. Translated reaction mixtures (10 μL) were run in a Tricine‐SDS PAGE gel and imaged with a trans‐UV 302 nm excitation light source. In‐gel fluorescence was observed for the translation sample with 4CNW, but not with tryptophan (Figure 2d) confirming that 4CNW is a highly sensitive fluorophore that can be detected in low quantities, such as those present in a 10 μL *in vitro* translation mixture. In‐gel visualisation of AzAla was not attempted due to a suitable excitation light source being unavailable.^[15]^

Finally, we assessed the potential of using either 4CNW or AzAla for biological imaging of peptides. Gai and co‐workers have previously demonstrated 4CNW as a satisfactory fluorophore for imaging by coupling to an antimicrobial peptide.^[9]^ However, to the best of our knowledge, the use of AzAla in fluorescence microscopy has not yet been explored. To evaluate their suitability for tracking peptide penetration into cells, 4CNW/AzAla were appended onto the *N*‐terminus of a well‐characterized cell penetrating peptide, TAT‐NLS **P3** (tryptophan was included as a negative control). Human bone osteosarcoma epithelial cells (U2OS) were incubated with peptides **4CNW**‐**P3**, **AzAla**‐**P3**, **W**‐**P3** for 20 minutes at a concentration of 50 μM in OPTI‐MEM. A moderate concentration (50 μM) of peptide was required for detection due to current limitations in the sensitivity of our microscopy system. Cells were then washed once with OPTI‐MEM and imaged on a Nikon Ti Eclipse inverted microscope with fluorescence excitation at 340 nm. An ET460/50 m single bandpass emission filter was used to image **4CNW**‐**P3** and a ET395/25x single bandpass emission filter was used to image **AzAla**‐**P3**. Our images show considerable intracellular fluorescence suggesting 4CNW and AzAla are suitable fluorophores for evaluating cell permeability of peptides when coupled with fluorescence microscopy (Figure 3a). We noted significant levels of cytotoxicity for all three TAT‐NLS peptides over longer incubation periods (e. g. 2 h), as measured by the release of lactate dehydrogenase (Figure S6). We envisage the cytotoxicity may be attributed to the combination of the TAT‐NLS sequence and the additional *N*‐terminal appendage, such that the evaluation of cell viability must be considered when combining these fluorescent amino acids with novel peptide sequences.

**Figure 3:**
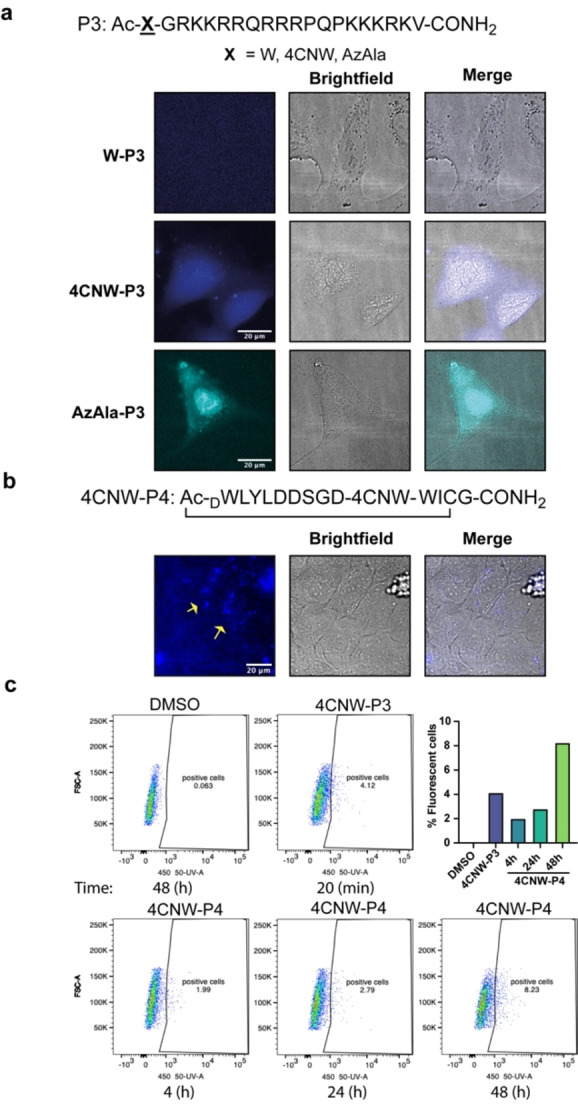
**Uptake of 4CNW/AzAla labelled peptides by living cells. a** Live cell fluorescence microscopy images of U2OS cells after 20 min incubation with linear/cyclic peptides (50 μM). Fluorescence of peptides is shown in blue (4CNW) or cyan (AzAla). Scale bar: 20 μm. Images were obtained by using fluorescence excitation at 340 nm with a ET460/50 m (4CNW) or ET395/25x (AzAla) single bandpass emission filter and a Plan Fluor 60x/A1.2 WI. **b** Live cell fluorescence microscopy images of U2OS cells after 24 h incubation with 4CNW‐P4 peptide (50 μM). Fluorescence of peptides is shown in blue (4CNW). Scale bar: 20 μm. Images were obtained by using fluorescence excitation at 340 nm with a ET460/50 m (4CNW) single bandpass emission filter and a Plan Fluor 40X/1.3NA objective. **c** Flow cytometry of U2OS cells incubated with DMSO, 4CNW‐P3 or 4CNW‐P4 at the indicated times. Fluorescence of a single cell suspension was acquired with a 355 nm laser, 450‐50 detector and FACS‐Diva software. Numbers indicated in graphs show percentage of positive cells.

To validate the use of 4CNW in studying the cell penetration of *de novo* cyclic peptides, we also substituted 4CNW for an internal tryptophan residue in a recently reported K48 Ub chain‐binding cyclic peptide **P4**, identified using the RaPID system.^[16]^ To assess if **4CNW**‐**P4** was penetrating cells, U2OS cells were incubated with 50 μM peptide for 24 h. Cells were then imaged by fluorescence microscopy using the same conditions as described above. As shown in Figure 3b, appreciable intracellular fluorescence was observed suggesting efficient uptake of the cyclic peptide, consistent with data previously reported.^[16]^

Flow cytometry has been widely used to study internalization of fluorescently tagged peptides in cells. Therefore, we wanted to explore the use of 4CNW for monitoring cellular uptake of peptides by flow cytometry. U2OS cells were incubated with 50 μM of **4CNW**‐**P3** or **4CNW**‐**P4** at different time points and the fluorescence of a single cell solution was measured. After 4 h incubation with **4CNW**‐**P4** we could detect a small percentage of positive blue fluorescent cells and this percentage was slightly increased at longer incubation times (Figure 3c, Figure S7). Incubation of cells with **4CNW**‐**P3** for 20 minutes was sufficient for initiating the detection of positive cells. These data indicate that flow cytometry is a good technique for monitoring the uptake of peptides containing 4CNW.

Label‐free assays are the gold standard for determining the cell permeability of cyclic peptide leads. Here, we report the incorporation of ClAc−4CNW and ClAc−AzAla into *in vitro* translated macrocyclic peptides *via* initiator reprogramming. In addition, we demonstrate 4CNW and AzAla are good tools for tracking peptide (linear/cyclic) penetration into cells. RaPID selections for *de novo* cyclic peptides initiated with ClAc−4CNW or ClAc−AzAla will enable the identification of peptide leads that are inherently fluorescent and can be directly assessed for cell permeability by fluorescence microscopy and flow cytometry. 4CNW and AzAla have also recently been used to probe protein‐protein binding interactions by PET, FRET and VET.[^9^, ^17^, ^18^] We therefore also envisage that 4CNW/AzAla initiated peptide leads could be used directly in label‐free binding assays.

## Conflict of interest

The authors declare no conflict of interest.

## Supporting information

As a service to our authors and readers, this journal provides supporting information supplied by the authors. Such materials are peer reviewed and may be re‐organized for online delivery, but are not copy‐edited or typeset. Technical support issues arising from supporting information (other than missing files) should be addressed to the authors.

Supporting Information
