# Supplementary material for: Fluorescent Amino Acid Initiated de novo Cyclic Peptides for the Label‐Free Assessment of Cell Permeability
Source: ChemMedChem. 2021 Jul 28;16(20):3185–8. doi: 10.1002/cmdc.202100315 (PMC8597039; doi:10.1002/cmdc.202100315)

ChemMedChem

Supporting Information

# Fluorescent Amino Acid Initiated *de novo* Cyclic Peptides for the Label-Free Assessment of Cell Permeability**

Yuteng Wu[+], M. Teresa Bertran[+], James Rowley, Ewen D. D. Calder, Dhira Joshi, and Louise J. Walport*

# Fluorescent amino acid initiated *de novo* cyclic peptides for the label-free assessment of cell permeability

## Supporting information

Yuteng Wu,† [a,b] M. Teresa Bertran,† [b] James Rowley,[a,b] Ewen D. D. Calder,[a,b] Dhira Joshi,[c] and Louise J. Walport*[a,b]

a. Protein-Protein Interaction Laboratory, The Francis Crick Institute, London NW1 1AT, United Kingdom

b. Department of Chemistry, Molecular Sciences Research Hub, Imperial College London, London W12 0BZ, United Kingdom

c. Peptide Chemistry, The Francis Crick Institute, London NW1 1AT, United Kingdom

† These authors contributed equally to this work

Email address: Louise.walport@crick.ac.uk

**Table of contents**


1. Synthetic procedures

2. Aminoacylation of microhelix RNA and tRNA

3. Translation and MALDI-TOF mass spectrometry of model peptides

4. Fluorescence visualization of translated peptide

5. Cell culture and fluorescence microscopy

6. LDH leakage toxicity assay

7. Flow cytometry

# *S1. Synthetic procedures*

## *S1.1. General chemistry experimental*

**Reagents and solvents** were purchased from Acros Organics, Alfa Aesar, Apollo Scientific, Fisher Scientific, Fluka, Fluorochem, Merck or Sigma Aldrich and were used without further purification. **Lyophilization** was carried out using a VirTis BenchTop Pro freeze dryer (8.0 L, –105 ˚C). **Normal and reverse phase chromatography** were performed on a Biotage (Uppsala, Sweden) Isolera One equipped with Biotage cartridges (SNAP KP-SIL, SNAP ULTRA or Sfär). **Nuclear magnetic resonance** spectra were recorded on a Bruker AV-400 spectrometer with the stated solvents as a reference for the internal deuterium lock. Chemical shifts are reported as $\delta_H$ or $\delta_C$ in parts per million (ppm) relative to tetramethylsilane (TMS). The spectra are calibrated using the solvent peak with the data provided by Fulmer *et al.*[1] **$^1$H NMR spectra,** Identical proton coupling constants are averaged in each spectrum and reported to the nearest 0.1 Hz. Coupling constants (J) are given in Hz to the nearest 0.1 Hz. Data are reported as follows: chemical shift multiplicity (s = singlet, d = doublet, t = triplet, q = quartet, m = multiplet and br = broad). Signals were assigned by the analysis of the chemical shifts, coupling and $^1$H-$^1$H COSY, $^{13}$C-$^1$H HSQC and $^{13}$C-$^1$H HMBC. The coupling constants were determined by analysis using Mestrenova software. **$^{13}$C NMR spectra** were recorded in the stated solvents with broadband proton decoupling and an internal deuterium lock. The shift values of resonances are quoted to 1 decimal place unless peaks have similar chemical shifts, in which case 2 decimal places are used. Signals were assigned by the analysis of the chemical shifts, $^{13}$C-$^1$H HSQC and $^{13}$C-$^1$H HMBC. **Purity** was determined by LC-MS and all tested compounds were of >95% purity. **LC-MS** data were obtained on a Waters ACQUITY (Massachusetts, USA) equipped with QSM, QDa and PDA detectors, sample manager FTN-H, quaternary solvent manager, column manager with ACQUITY UPLC BEH C18 1.7 µm, 2.1 x 50 mm column. Electrospray ionization (ES+ and ES-) and Diode Array spectra were obtained for each characterised compound. The gradient method for LC-MS was 95%-5% 0.1% formic acid (FA) in water/ 0.1 % FA in acetonitrile (MeCN/ACN), over 4 minutes, 0.5 ml/min, 1 µL injection.

## *S1.2. Synthesis of ClAc-CNW-CME and ClAc-AzAla-CME*

4-Cyanotryptophan (4CNW) **3**, β-(1-Azulenyl)-L-Alanine (AzAla) **4** were prepared according to previously described protocols.[2] Fmoc-4CNW and Fmoc-AzAla were obtained from **3** and **4** following the literature procedure.[3]

### ***N*-Chloroacetyl 4-cyanotryptophan (ClAc-4CNW)**

*N*-(Chloroacetoxy) succinimide (60 mg, 0.31 mmol) in THF (2 mL) was added to a stirring suspension of 4CNW **3** (50 mg, 0.22 mmol) in aqueous $Na_2CO_3$ (0.1 M, 4 mL) at rt. The reaction mixture was stirred for 1 h at rt. THF was removed *in vacuo* and the mixture was acidified to pH 2 by the addition of 1 M aqueous hydrochloric acid. The mixture was extracted with dichloromethane (3 x 30 mL). The combined organic phase was washed with water (10 mL), dried over $Na_2SO_4$ and concentrated *in vacuo*. The residue was purified by reverse-phase column chromatography (acetonitrile with 0.5% formic acid/water with 0.5% formic acid, 0-80%) to give the title compound as a colourless solid (46 mg, 0.15 mmol, 69%). $^1$H NMR (400 MHz, [D6]DMSO): $\delta$= 11.56 (d, *J*=2.2, 1H), 8.53 (d, *J*=8.2, 1H), 7.70 (dd, *J*=8.2, 0.9, 1H), 7.48 (dd, *J*=7.4, 0.9, 1H), 7.39 (d, *J*=2.2, 1H), 7.21 (dd, *J*=8.2, 7.4, 1H), 4.59 (ddd, *J*=9.2, 8.2, 4.9, 1H), 4.05 (s, 2H), 3.47 (dd, *J*=15.3, 4.9, 1H), 3.25 (dd, *J*=15.3, 9.2, 1H); $^{13}$C NMR (101 MHz, [D6]DMSO): $\delta$=172.7, 165.7, 136.3, 127.4, 126.0, 125.5, 120.8, 119.3, 117.0, 109.4, 100.2, 52.9, 42.4, 26.5; LCMS: rt 1.03 min, purity >99%, *m/z* (ESI$^+$) 308.2 ([MH]$^+$, 30%), 306.2 ([MH]$^+$, 100%), (ESI$^-$) *m/z* 609.1 ([2M-H]$^-$, 100%), 611.2 ([2M-H]$^-$, 75%), 306.2 ([M-H]$^-$, 20%), 308.2 ([M-H]$^-$, 60%).

**N-Chloroacetyl 4-cyanotryptophan cyanomethyl ester (ClAc-4CNW-CME)**

Triethylamine (15 µL, 0.11 mmol) was added to a stirring solution of ClAc-4CNW **5** (15 mg, 49 µmol) in acetonitrile/chloroacetonitrile (1:1, 1 mL) at rt. The reaction mixture was stirred for 16 h at rt. The solvent was removed *in vacuo*. The residue was purified by silica column chromatography (ethyl acetate/petroleum ether, 0-80%) to give the title compound as a colourless solid (13 mg, 38 µmol, 77%). $^{1}$H NMR (400 MHz, [D6]DMSO): $\delta$= 11.62 (d, *J*=2.6, 1H), 8.87 (d, *J*=7.3, 1H), 7.72 (dd, *J*=8.2, 0.9, 1H), 7.51 (dd, *J*=7.4, 0.9, 1H), 7.43 (d, *J*=2.6, 1H), 7.23 (dd, *J*=8.2, 7.4, 1H), 4.99 (s, 2H), 4.71 (ddd, *J*=9.0, 7.3, 6.1, 1H), 4.08 (s, 2H), 3.47 (dd, *J*=15.0, 6.1, 1H), 3.32 (dd, *J*=15.0, 9.0, 1H); $^{13}$C NMR (101 MHz, [D6]DMSO): $\delta$= 170.3, 166.2, 136.4, 128.1, 125.8, 125.6, 121.0, 119.3, 117.2, 115.5, 108.2, 100.0, 53.0, 49.5, 42.1, 26.0; LCMS: rt 2.01 min, purity 97%, *m/z* (ESI$^{+}$) 347.1 ([MH]$^{+}$, 30%), 345.1 ([MH]$^{+}$, 100%), (ESI$^{-}$) *m/z* 345.1 ([M-H]$^{-}$, 30%), 343.1 ([M-H]$^{-}$, 100%).

**N-Chloroacetyl 3-(azulen-1′-yl) alanine (ClAc-AzAla)**

*N*-(Chloroacetoxy) succinimide (60 mg, 0.31 mmol) in THF (2 mL) was added to a stirring suspension of AzAla **4** (50 mg, 0.23 mmol) in aqueous $Na_2CO_3$ (0.1 M, 4 mL) at rt. The reaction mixture was stirred for 1 h at rt. THF was removed *in vacuo* and the mixture was acidified to pH 2 by the addition of 1 M aqueous hydrochloric acid. The mixture was extracted with dichloromethane (3 x 30 mL). The combined organic phase was washed with water (10 mL), dried over $Na_2SO_4$ and concentrated *in vacuo*. The residue was purified by reverse-phase column chromatography (acetonitrile with 0.5 % formic acid/water with 0.5 % formic acid, 0-80%) to give the title compound as a blue solid (48 mg, 0.16 mmol, 71%). $^{1}$H NMR (400 MHz, [D6]DMSO): $\delta$= 8.49 (d, *J*=7.9, 1H, NH), 8.36 (d, *J*=9.7, 1H), 8.33 (dd, *J*=9.7, 1.2, 1H), 7.80 (d, *J*=3.8, 1H), 7.64 (dddd, *J*=9.8, 9.8, 1.2, 1.1, 1H), 7.33 (d, *J*=3.8, 1H), 7.19 (dd, *J*=9.8, 9.7, 1H), 7.16 (dd, *J*=9.8, 9.7, 2H), 4.57 (ddd, *J*=8.0, 7.9, 5.2, 1H), 4.09 – 4.01 (m, 2H), 3.57 (dd, *J*=14.6, 5.2, 1H), 3.43 (dd, *J*=14.6, 8.0, 2H); $^{13}$C NMR (101 MHz, [D6]DMSO): $\delta$= 172.7, 165.8, 140.3, 137.9, 137.7, 136.6, 136.2, 133.6, 125.2, 122.6, 122.1, 116.7, 54.1, 42.3, 28.8; LCMS: rt 2.25 min, purity >99%, (ESI$^{+}$) *m/z* 292.1 ([MH]$^{+}$, 100%), (ESI$^{-}$) *m/z* 290.1 ([M-H]$^{-}$, 100%).

**N-Chloroacetyl 3-(azulen-1′-yl) alanine cyanomethyl ester (ClAc-AzAla-CME)**

Triethylamine (15 µL, 0.11 mmol) was added to a stirring solution of ClAc-AzAla **6** (20 mg, 69 µmol) in acetonitrile/chloroacetonitrile (1:1, 1 mL) at rt. The reaction mixture was stirred for 16 h at rt. The solvent was removed *in vacuo*. The residue was purified by silica column chromatography (ethyl acetate/petroleum ether, 0-80%) to give the title compound as a blue solid (18 mg, 55 µmol, 79%). $^{1}$H NMR (400 MHz, [D6]DMSO): $\delta$= 8.84 (d, *J*=7.5, 1H), 8.36 (d, *J*=9.7, 1H), 8.35 (dd, *J*=9.7, 1.2, 1H), 7.81 (d, *J*=3.8, 1H), 7.67 (dddd, *J*=10.0, 9.9, 1.2, 1.1, 1H), 7.34 (d, *J*=3.8, 1H), 7.22 (dd, *J*=10.0, 9.7, 1H), 7.18 (dd, *J*=9.9, 9.7, 1H), 5.03 – 4.91 (m, 2H), 4.70 (ddd, *J*=8.7, 7.5, 5.9, 1H), 4.05 (s, 2H), 3.58 (dd, *J*=14.7, 5.9, 1H), 3.50 (dd, *J*=14.7, 8.7, 1H); $^{13}$C NMR (101 MHz, [D6]DMSO): $\delta$= 170.3, 166.2, 140.4, 138.0, 137.6, 136.7, 136.2, 133.6, 124.1, 122.8, 122.3, 116.8, 115.6, 53.8, 49.5, 42.1, 28.3; LCMS: rt 2.52 min, >99%, (ESI$^{+}$) *m/z* 331.2 ([MH]$^{+}$, 90%), 142.2 (100%), (ESI$^{-}$) *m/z* 329.1 ([M-H]$^{-}$, 100%).

## ***S1.3. Peptide synthesis***

Peptide synthesis was performed on solid-phase using standard Fmoc-protecting group strategy on an Intavis ResPep SLi automated synthesizer (Intavis Bioanalytical Instruments AG, Cologne Germany) using Rink Amide AM resin LL (0.05 mmol/g, Merck). All peptide couplings were performed with Fmoc-protected amino acids (5 equiv) in DMF, HATU (5 equiv) in DMF, and *N*,*N*-diisopropylethylamine (10 equiv) in NMP. Fmoc deprotection was carried out with 20 % piperidine in DMF. Couplings with Fmoc 4CNW/AzAla were carried out manually with Fmoc-protected amino acids (1.3 equiv), DIC/Oxyma (1.3 equiv.), *N*,*N*-diisopropylethylamine (1.3 equiv) in NMP. *N*-terminal capping was performed manually by treating the resin-bound peptide with 20% acetic anhydride in DMF or *N*-(chloroacetoxy)succinimide in DMF for 1 h.

Cleavage was achieved with a cocktail of trifluoroacetic acid (92.5%), triisopropylsilane (2.5%), water (2.5%), 1,2-ethanedithiol (2.5%) for 2 h. The cleavage solution was then evaporated under a stream of nitrogen. The crude residue was triturated with diethyl ether prior to purification by HPLC, using a reversed phase preparative C8 column (Agilent PrepHT Zorbax 300SB-C8, 21.2x250 mm, 7 m) applying a flow rate of 8 mL/min and a linear gradient of 10 to 50% (v/v) solvent B for 40 min [solvent A: 99.9% (v/v) water and 0.1% (v/v) trifluoroacetic acid; solvent B: 99.9% (v/v) acetonitrile and 0.1% (v/v) trifluoroacetic acid]. The purified peptides were analyzed on an Agilent 1100 LC-MSD system.

Peptide cyclisation was carried out by incubating the linear peptide (< 1 mg/mL) in aqueous buffer containing ammonium solution (0.25 M, pH 7-8). The reaction mixture was shaken for 1 h, lypohilized and purified by HPLC to give the final cyclic peptide.

## ***S1.4. Peptide LCMS data***

The *m/z* ratios show the $[M+3H]^{3+}$ species unless otherwise stated.

| Peptide | *m/z* found | *m/z* calcd |
|---|---|---|
| P3 | 843.3 | 843.2 |
| W-P3 | 905.3 | 905.2 |
| 4CNW-P3 | 914.0 | 913.9 |
| AzAla-P3 | 909.0 | 909.2 |
| 4CNW-P4 | 897.0 $[M+2H]^{2+}$ | 896.9 $[M+2H]^{2+}$ |

Table S1.4.1. LCMS data for linear and cyclic peptides.

## ***S1.5. mRNA template synthesis***

The mRNA templates 1 and 2 used in this study were constructed by two rounds of overlapping PCR. In brief, the first round of PCR was done at 100 µL scale (1X KOD polymerase buffer, 1 mM $MgCl_2$, 0.2 mM dNTPs, 0.6 µM T7g10M.F46 primer, 0.5 µM Primer 1 or 2, 0.8 µL KOD polymerase) for 5 cycles and an annealing temperature of 55 °C. The second round of PCR was done at 200 µL scale using the products of the first round as templates (1X KOD buffer, 1 mM $MgCl_2$, 0.1 mM dNTPs, 0.25 µM T7g10M.F46 primer, 0.25 µM CGS3an13.R39 primer) with an annealing temperature of 61 °C for 4 cycles.

The PCR product was purified by phenol-chloroform extraction followed by ethanol precipitation. The purified product was then transcribed overnight using T7 RNA polymerase (Thermo Scientific) following the manufacturer's protocol. The RNA was isolated by isopropanol precipitation and further purified by urea denaturing 8% PAGE gel.

| Oligo ID | Sequence |
|---|---|
| T7g10M.F46 | TAATACGACTCACTATAGGGTTAACTTTAAGAAGGAGATATACATA |
| CGS3an13.R39 | TTTCCGCCCCCCGTCCTAGCTGCCGCTGCCGCTGCCGCA |
| Primer 1 (Template 1) | GCTGCCGCTGCCGCTGCCGCAAAGACGAAGCACCCGCTGATACTGACACT CCGACATATGTATATCTCCTTCTTAAAG |
| Primer 2 (Template 2) | AAGAAGGAGATATACATATGAAAACCATTATGGGCATGACCTGGCGCACC ATGCAGTGCGGCAGCGGCAGCGGC |

Table S1.5.1. List of oligonucleotides used in this work.

## S2. Aminoacylation of microhelix RNA and tRNA

Aminoacylation was performed by mixing 5 mM CME substrates **8** or **9** with 600 mM $MgCl_2$, 20% DMSO, 25 µM eFx, 25 µM microhelix (FAM-MiHx_23b, 5'- /56-FAM/rArGrG rCrUrC rUrGrU rUrCrG rCrArG rArGrC rCrGrC rCrA -3', Integrated DNA Technologies) or initiator tRNA, 50 mM HEPES-KOH (pH 7.5 or 9.0). The mixture was incubated for 2, 4, 8 or 16 h on ice. Flexizyme eFx and initiator tRNA were synthesised according to the previously described protocol.[4] The resulting aminoacyl-microhelix/tRNA was purified by ethanol precipitation. The pellets were washed with 2x 70% ethanol containing 0.1 M sodium acetate (pH 5.2), and analyzed on a 20% polyacrylamide gel containing 50 mM sodium acetate (pH 5.2) by detection of the FAM label on a Typhoon FLA 9500 (GE Healthcare) and quantified with Fiji.[5]

## S3. Translation and MALDI-TOF mass spectrometry of model peptides

Translation of model peptides **P1** was performed using a PURExpress$^{TM}$ Δ (aa, tRNA) *in vitro* protein synthesis kit (NEB) according to the manufacture's protocol. Translation mixtures were prepared on ice by combining 1.0 µL solution A, 1.5 µL solution B, 0.5 µL tRNA, 0.5 µL aminoacyl-tRNA (prepared as described above S2., pH 9.0, 2 h), 0.5 µL mRNA template (Template 1, 10 µM), 0.5 µL amino acid mixture (-Met), 0.5 µL water. The translation reaction mixture was incubated at 37 °C for 1 h. The resulting mixture was desalted and concentrated with $ZipTip_{u\text{-}c18}$ (Millipore), co-crystallised with α-cyano-4-hydroxycinnamic acid and analyzed in positive mode using Micromass MALDI-TOF (Waters).

Translation of model peptides **P2** was performed using a PURExpress$^{TM}$ Δ (aa, tRNA) *in vitro* protein synthesis kit (NEB) according to the manufacture's protocol. Translation mixtures were prepared on ice by combining 1.0 µL solution A, 1.5 µL solution B, 0.5 µL tRNA, 0.5 µL mRNA template (Template 2, 10 µM), 0.5 µL amino acid mixture (-Trp, supplemented with **3** or **4**), 1 µL water. The translation reaction mixture was incubated at 37 °C for 1 h and analyzed MALDI-TOF mass spectrometry as described above.

## S4. Fluorescence visualization of translated peptide

*In vitro* translation reactions expressing peptides **CNW-P1, W-P1** were carried out as described above S3. To 10 µL of translated mixture was added 4X Laemli Sample buffer to terminate translation and the resulting mixture were run on a 15% tricine-SDS-PAGE gel as previously described.[6] In gel fluorescence was imaged in a Chemidoc MP Imaging System (Biorad) using stain free conditions (trans-UV 302 nm excitation).

## S5. Cell culture and fluorescence microscopy

Human bone osteosarcoma epithelial cells (U2OS, Crick Cell Services) were cultured in 5% $CO_2$ atmosphere and 37 °C in DMEM (Dulbecco's Modified Eagle's Medium, GIBCO) supplemented with 10% FBS (Fetal Bovine Serum, Sigma Aldrich) and Penicillin/Streptomycin (100 µg/mL, GIBCO). Cells were seeded in an 8 well glass bottom µ-Slide (Ibidi) at a density of 200000 cell/well the day before the experiment. The following day, medium was aspirated and 100 µL of OPTI-MEM (GIBCO) was added in each well. Peptides were dissolved in DMSO and diluted to 250 µM in OPTI-MEM to a final DMSO concentration of 2.5%. 25 µL peptide was added to each well to achieve a final peptide concentration of 50 µM and the cells were incubated at 37˚C with 5% $CO_2$ for 20 or 1440 min (final

DMSO concentration 0.5%). After incubation, cells were washed once with OPTI-MEM and imaged in phenol-red free DMEM (GIBCO). Widefield imaging was performed using a Ti Eclipse inverted microscope (Nikon) with motorised XY stage (ASI), using a Plan Fluor 60x/A1.2 WI or Plan Fluor 40x/1.3 NA objective and an Evolve EMCCD camera (Photometrics). The microscope was controlled with Micro-Manager v2.0 gamma software.[7] Fluorescence excitation at 340 nm was performed using a Fura-2 LED light engine (Cairn), a 400 longpass dichroic mirror (T400LP, Chroma) and ET460/50m single bandpass emission filter (Chroma) for 4CNW imaging and a ET395/25X single bandpass emission filter (Chroma) for AzAla imaging. Images were processed with Fiji.[5]

## ***S6. LDH leakage toxicity assay***

The protocol was based on an LDH assay previously carried out on peptides.[8] U2OS cells were grown in an identical manner to the microscopy protocol (see S5.). Peptides were added to cells, and after 20 min or 2 h, LDH leakage into cell media was analyzed using the CytoTox 96 Non-Radioactive Cytotoxicity Assay Kit (Promega G1780) according to the manufacturer's protocol. All controls (maximum LDH, vehicle control, cell-free controls) and LDH leakage calculations were conducted as previously reported.[8] Experiments were conducted in triplicate.

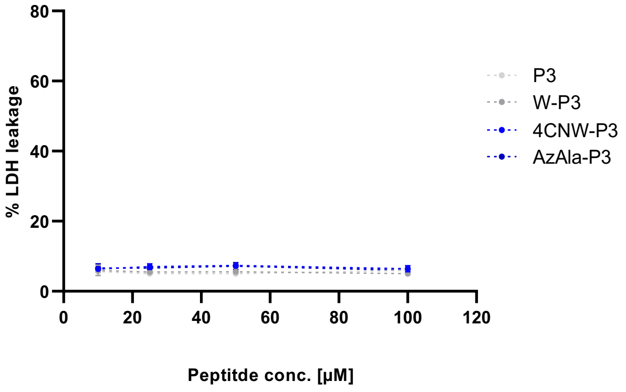


Figure S6.1. LDH leakage of U2OS cells when incubated for 20 min with up to 100 µM of peptide, as a measure of non-specific toxicity. Error bars represent the standard deviation of triplicate experiments.

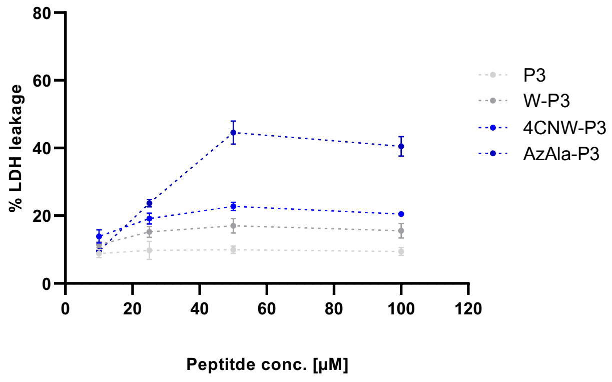


Figure S6.2. LDH leakage of U2OS cells when incubated for 2 h with up to 100 µM of peptide, as a measure of non-specific toxicity. Error bars represent the standard deviation of triplicate experiments.

## *S7. Flow cytometry*

U2OS cells were treated with 50 µM of the indicated peptides for 4, 24 or 48 h. 0.5% DMSO treated cells were used as negative control. After incubation cells were washed with PBS and trypsinized (Trypsin-EDTA 0.05% in PBS, Thermo Scientific). Single-cell suspensions were done in 350µM PBS with 1% FBS. Samples were acquired on a LSR-Fortessa (BD Bioscience) equipped with a 355 nm laser, 450-50 detector and FACS-Diva software and data was analyzed using FlowJo 10.3 software (Tree Star).

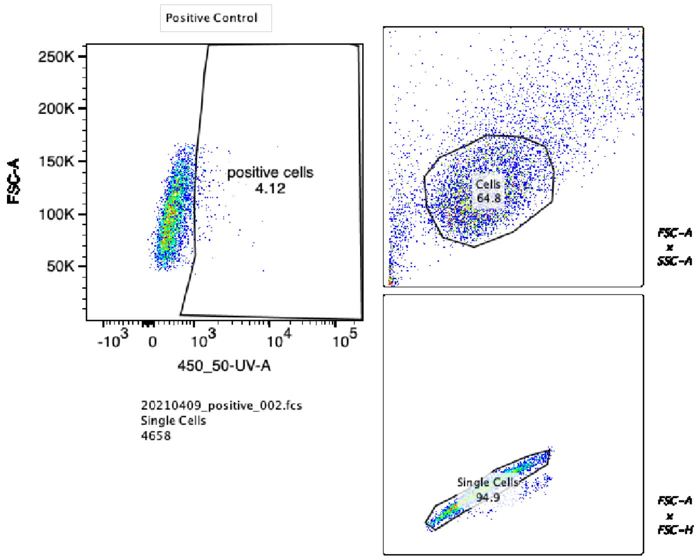


Figure S7. Example of the pipeline used to analyze the flow cytometry data. For all samples a gate was created to detect cells from the sample and a second gate allowed us to detect single cells. Fluorescence was measured on the single cell population (≈4000 single cells were acquired per condition).

## *References*


[1] G. R. Fulmer, A. J. M. Miller, N. H. Sherden, H. E. Gottlieb, A. Nudelman, B. M. Stoltz, J. E. Bercaw, K. I. Goldberg, *Organometallics* **2010**, *29*, 2176–2179.

[2] and F. H. A. Christina E. Boville,† David K. Romney,† Patrick J. Almhjell, Michaela Sieben, *J. Org. Chem.* **2018**, *83*, 7447–7452.

[3] K. Zhang, I. A. Ahmed, H. T. Kratochvil, W. F. Degrado, F. Gai, H. Jo, *Chem. Commun.* **2019**, *55*, 5095–5098.

[4] Y. Goto, T. Katoh, H. Suga, *Nat. Protoc.* **2011**, *6*, 779–790.

[5] J. Schindelin, I. Arganda-Carreras, E. Frise, V. Kaynig, M. Longair, T. Pietzsch, S. Preibisch, C. Rueden, S. Saalfeld, B. Schmid, J. Y. Tinevez, D. J. White, V. Hartenstein, K. Eliceiri, P. Tomancak, A. Cardona, *Nat. Methods* **2012**, *9*, 676–682.

[6] T. Kawakami, H. Murakami, H. Suga, *Chem. Biol.* **2008**, *15*, 32–42.

[7] A. D. Edelstein, M. A. Tsuchida, N. Amodaj, H. Pinkard, R. D. Vale, N. Stuurman, *J. Biol. Methods* **2014**, *1*, 10.

[8] Y. C. Li, L. W. Rodewald, C. Hoppmann, E. T. Wong, S. Lebreton, P. Safar, M. Patek, L. Wang, K. F. Wertman, G. M. Wahl, *Cell Rep.* **2014**, *9*, 1946–1958.

# ***NMR and LCMS spectra***

## *N*-Chloroacetyl 4-cyanotryptophan (ClAc-4CNW)

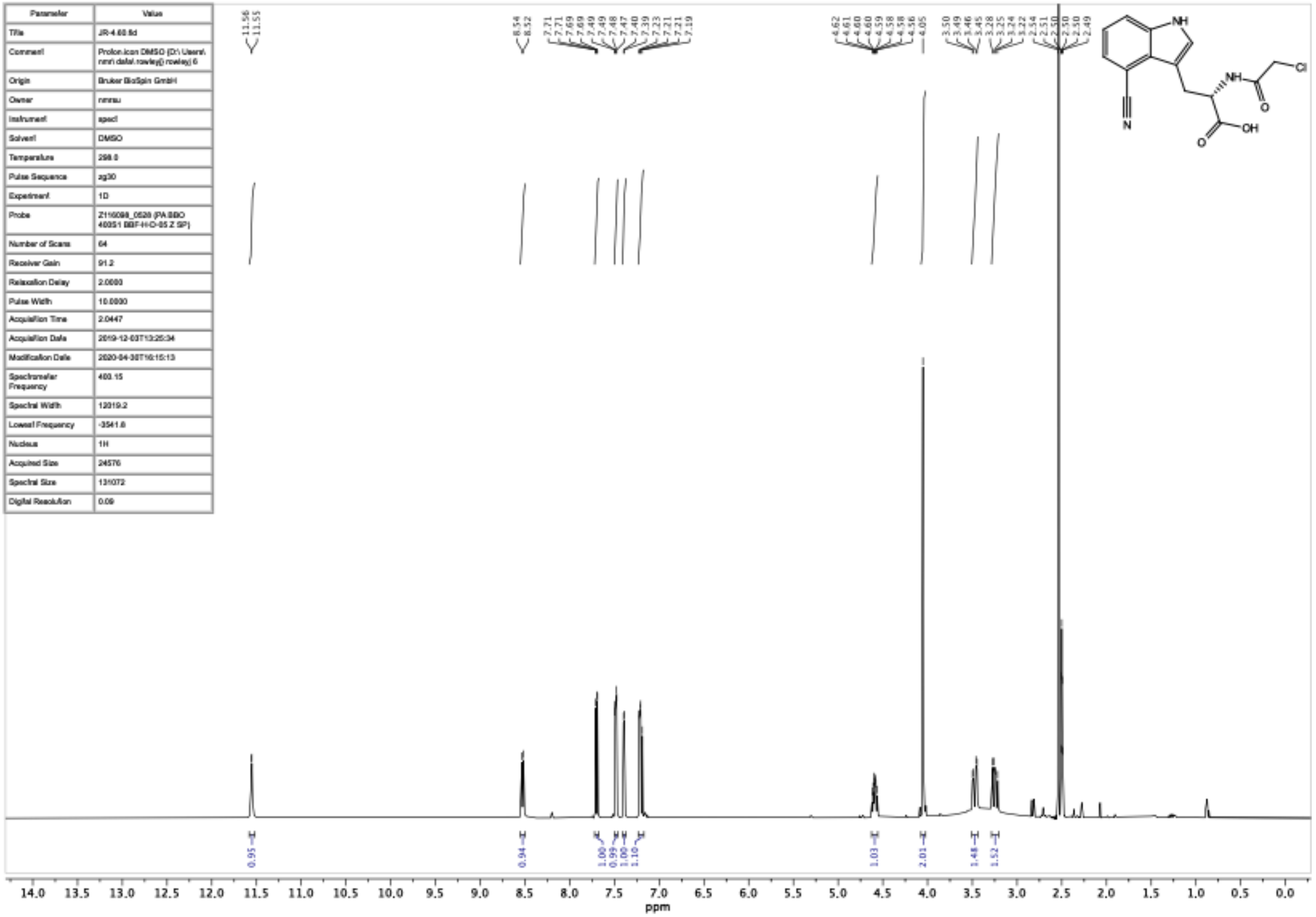


| Parameter | Value |
|---|---|
| Title | JR-4.60.fid |
| Comment | Prolon.icon DMSO (D:\ Users\ nmr\ data\ rowley() rowley) 6 |
| Origin | Bruker BioSpin GmbH |
| Owner | nmrsu |
| Instrument | spect |
| Solvent | DMSO |
| Temperature | 298.0 |
| Pulse Sequence | zg30 |
| Experiment | 1D |
| Probe | Z116098_0520 (PA BBO 400S1 BBF-H-D-05 Z SP) |
| Number of Scans | 64 |
| Receiver Gain | 91.2 |
| Relaxation Delay | 2.0000 |
| Pulse Width | 10.0000 |
| Acquisition Time | 2.0447 |
| Acquisition Date | 2019-12-03T13:25:34 |
| Modification Date | 2020-04-30T16:15:13 |
| Spectrometer Frequency | 400.15 |
| Spectral Width | 12019.2 |
| Lowest Frequency | -3541.6 |
| Nucleus | 1H |
| Acquired Size | 24576 |
| Spectral Size | 131072 |
| Digital Resolution | 0.09 |

# *N*-Chloroacetyl 4-cyanotryptophan (ClAc-4CNW)

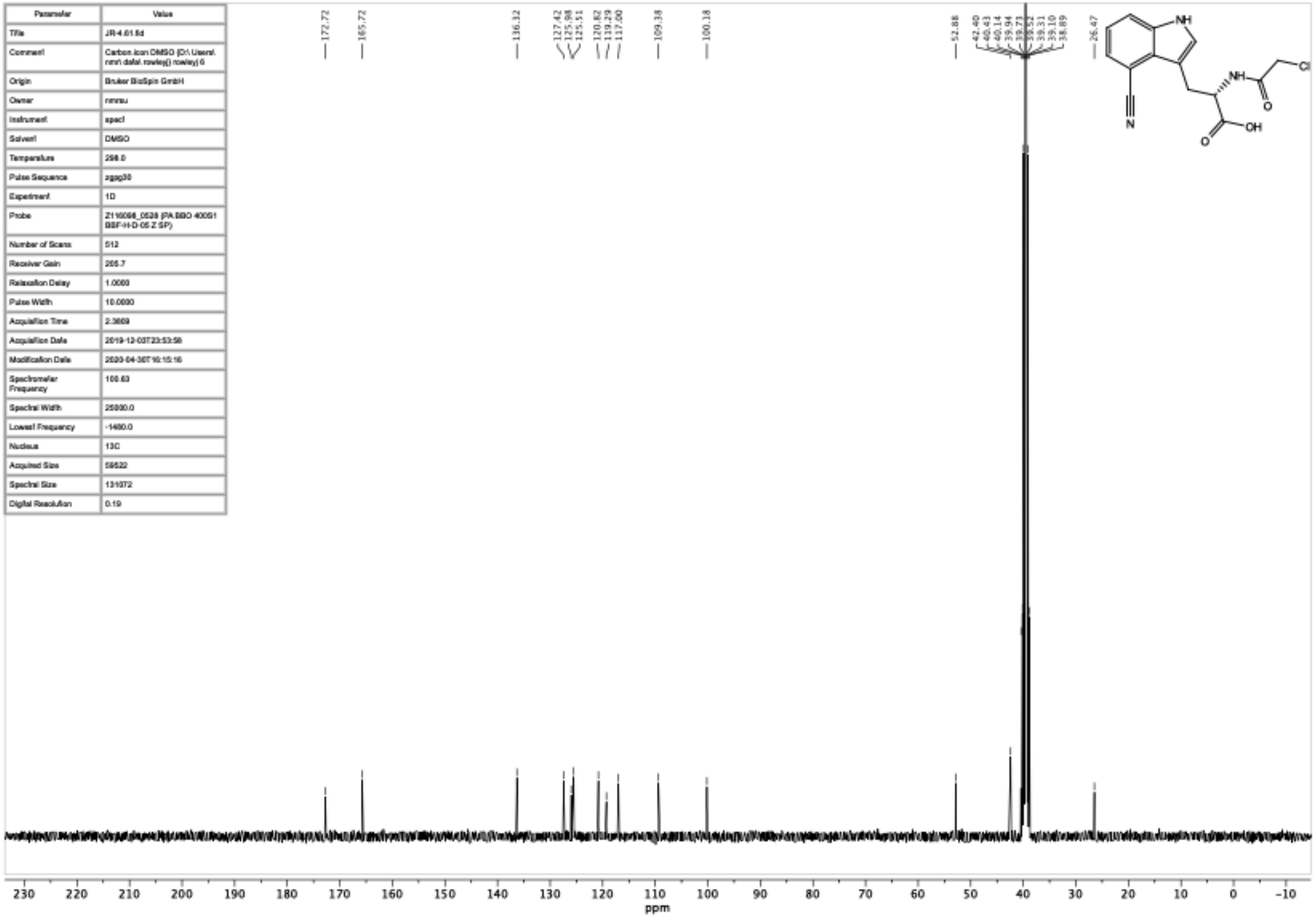


| Parameter | Value |
|---|---|
| Title | JR-4.61.fid |
| Comment | Carbon.icon DMSO {D:\ Users\ nmr\ data\ rowley(j) rowley} 6 |
| Origin | Bruker BioSpin GmbH |
| Owner | nmrsu |
| Instrument | spect |
| Solvent | DMSO |
| Temperature | 298.0 |
| Pulse Sequence | zgpg30 |
| Experiment | 1D |
| Probe | Z116098_0528 (PA BBO 400S1 BBF-H-D-05 Z SP) |
| Number of Scans | 512 |
| Receiver Gain | 205.7 |
| Relaxation Delay | 1.0000 |
| Pulse Width | 10.0000 |
| Acquisition Time | 2.3609 |
| Acquisition Date | 2019-12-03T23:53:58 |
| Modification Date | 2020-04-30T16:15:16 |
| Spectrometer Frequency | 100.63 |
| Spectral Width | 25000.0 |
| Lowest Frequency | -1480.0 |
| Nucleus | 13C |
| Acquired Size | 59522 |
| Spectral Size | 131072 |
| Digital Resolution | 0.19 |

## *N*-Chloroacetyl 4-cyanotryptophan cyanomethyl ester (ClAc-4CNW-CME)

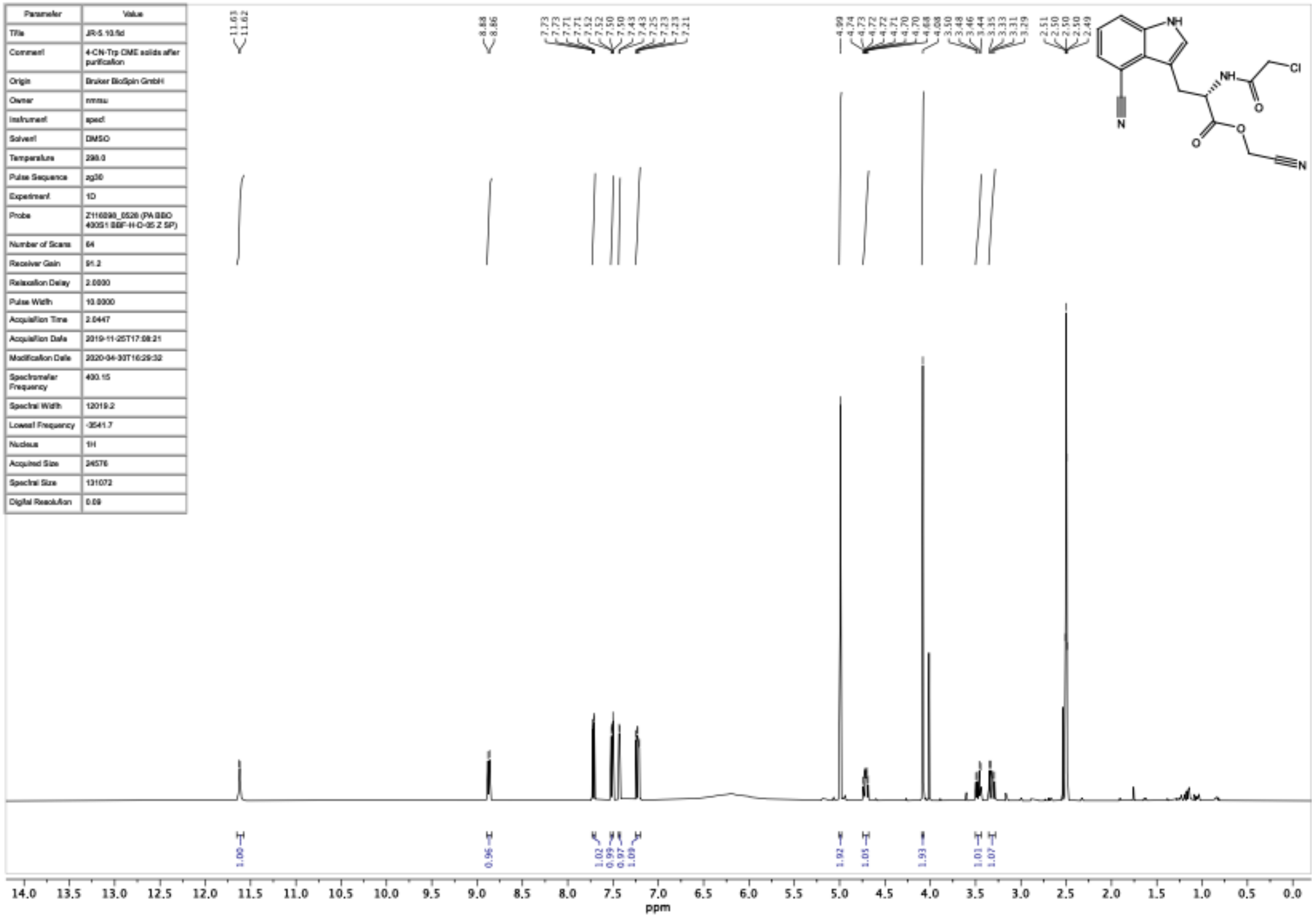


| Parameter | Value |
|---|---|
| Title | JR-5.10.fid |
| Comment | 4-CN-Trp CME solids after purification |
| Origin | Bruker BioSpin GmbH |
| Owner | nmrsu |
| Instrument | spect |
| Solvent | DMSO |
| Temperature | 298.0 |
| Pulse Sequence | zg30 |
| Experiment | 1D |
| Probe | Z116098_0526 (PA BBO 400S1 BBF-H-D-05 Z SP) |
| Number of Scans | 64 |
| Receiver Gain | 91.2 |
| Relaxation Delay | 2.0000 |
| Pulse Width | 10.0000 |
| Acquisition Time | 2.0447 |
| Acquisition Date | 2019-11-25T17:08:21 |
| Modification Date | 2020-04-30T16:29:32 |
| Spectrometer Frequency | 400.15 |
| Spectral Width | 12019.2 |
| Lowest Frequency | -3541.7 |
| Nucleus | 1H |
| Acquired Size | 24576 |
| Spectral Size | 131072 |
| Digital Resolution | 0.09 |

## *N*-Chloroacetyl 4-cyanotryptophan cyanomethyl ester (ClAc-4CNW-CME)

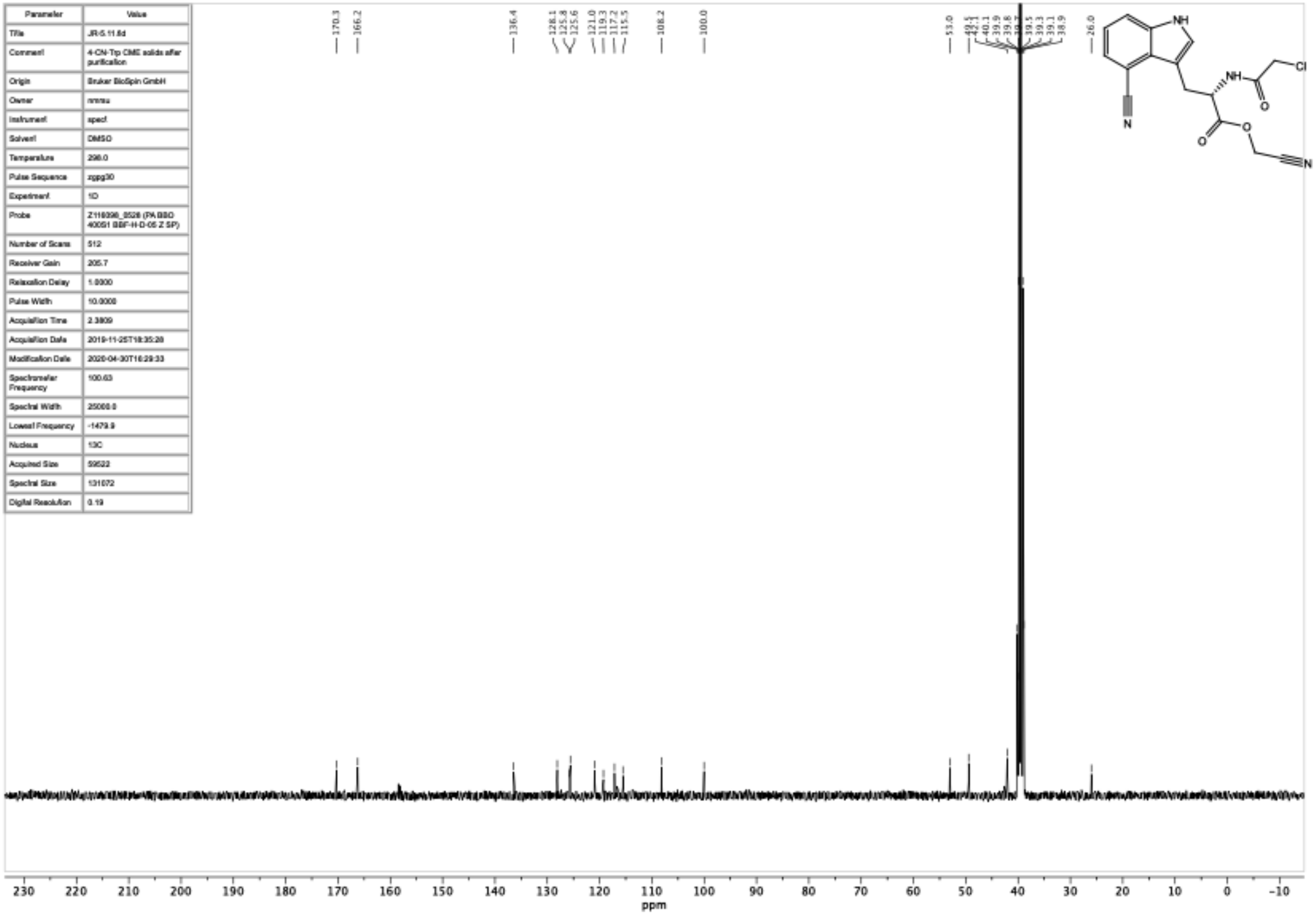


| Parameter | Value |
|---|---|
| Title | JR-5.11.8d |
| Comment | 4-CN-Trp CME solids after purification |
| Origin | Bruker BioSpin GmbH |
| Owner | nmrsu |
| Instrument | spect |
| Solvent | DMSO |
| Temperature | 298.0 |
| Pulse Sequence | zgpg30 |
| Experiment | 1D |
| Probe | Z116098_0528 (PA BBO 400S1 BBF-H-D-05 Z SP) |
| Number of Scans | 512 |
| Receiver Gain | 205.7 |
| Relaxation Delay | 1.0000 |
| Pulse Width | 10.0000 |
| Acquisition Time | 2.3809 |
| Acquisition Date | 2019-11-25T18:35:28 |
| Modification Date | 2020-04-30T16:29:33 |
| Spectrometer Frequency | 100.63 |
| Spectral Width | 25000.0 |
| Lowest Frequency | -1479.9 |
| Nucleus | 13C |
| Acquired Size | 59522 |
| Spectral Size | 131072 |
| Digital Resolution | 0.19 |

# *N*-Chloroacetyl 3-(azulen-1'-yl) alanine (ClAc-AzAla)

| Parameter | Value |
|---|---|
| Title | ClAc-Azu-OH.10.fid |
| Comment | ClAc-Azu-OH Proton.icon DMSO {D:\ Users\ nmr\ data\ caldere} caldere 43 |
| Origin | Bruker BioSpin GmbH |
| Owner | nmrsu |
| Instrument | spect |
| Solvent | DMSO |
| Temperature | 297.7 |
| Pulse Sequence | zg30 |
| Experiment | 1D |
| Probe | Z116098_0528 (PA BBO 400S1 BBF-H-D-05 Z SP) |
| Number of Scans | 64 |
| Receiver Gain | 91.2 |
| Relaxation Delay | 2.0000 |
| Pulse Width | 10.0000 |
| Acquisition Time | 2.0447 |
| Acquisition Date | 2021-02-23T23:41:48 |
| Modification Date | 2021-02-23T23:41:48 |
| Spectrometer Frequency | 400.15 |
| Spectral Width | 12019.2 |
| Lowest Frequency | -3538.5 |
| Nucleus | 1H |
| Acquired Size | 24576 |
| Spectral Size | 131072 |
| Digital Resolution | 0.09 |

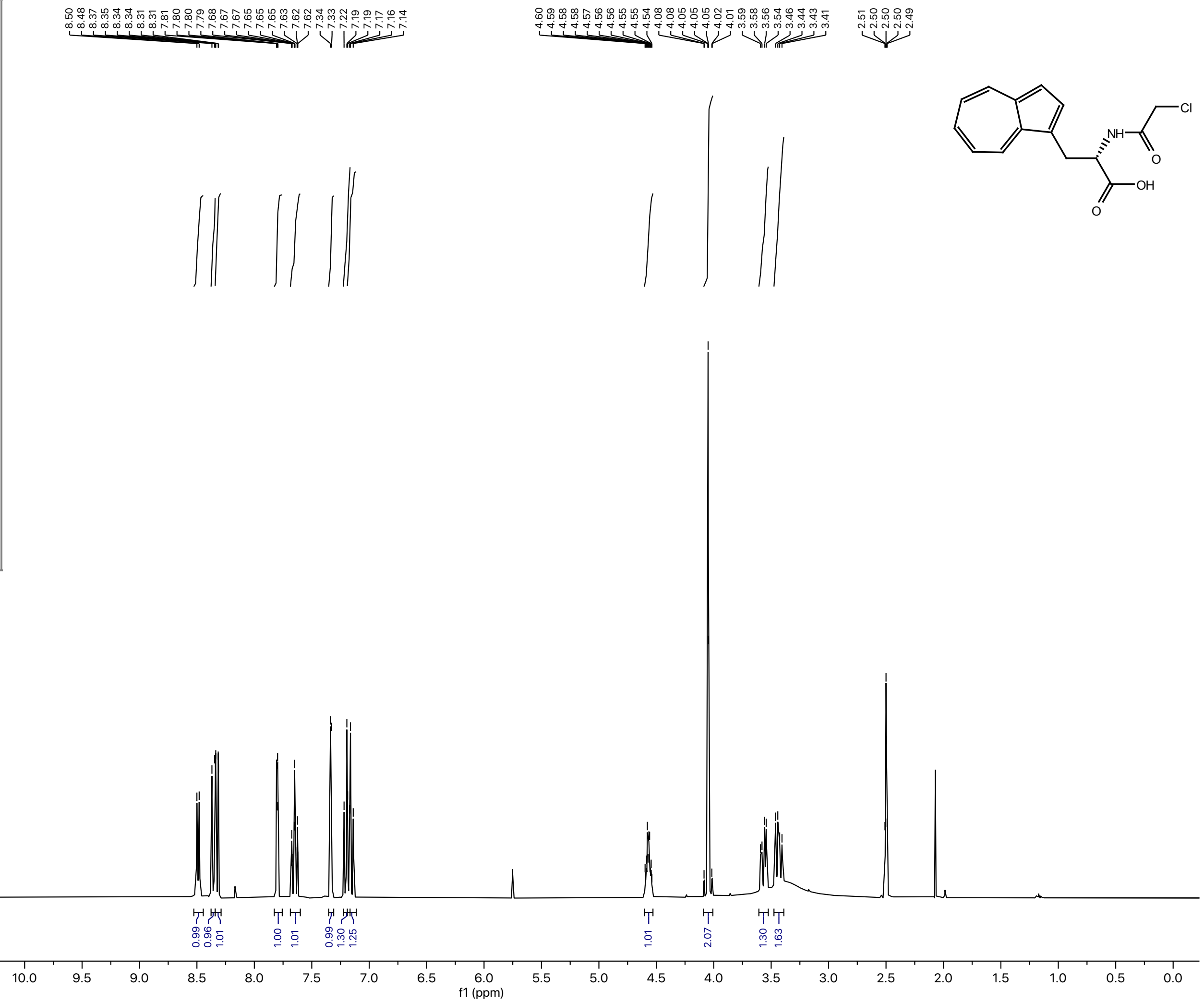

# *N*-Chloroacetyl 3-(azulen-1'-yl) alanine (ClAc-AzAla)

| Parameter | Value |
|---|---|
| Title | ClAc-Azu-OH.14.fid |
| Comment | ClAc-Azu-OH Carbon.icon DMSO {D:\ Users\ nmr\ data\ caldere} caldere 43 |
| Origin | Bruker BioSpin GmbH |
| Owner | nmrsu |
| Instrument | spect |
| Solvent | DMSO |
| Temperature | 297.7 |
| Pulse Sequence | zgpg30 |
| Experiment | 1D |
| Probe | Z116098_0528 (PA BBO 400S1 BBF-H-D-05 Z SP) |
| Number of Scans | 1024 |
| Receiver Gain | 205.7 |
| Relaxation Delay | 1.0000 |
| Pulse Width | 10.0000 |
| Acquisition Time | 2.3809 |
| Acquisition Date | 2021-02-24T02:06:28 |
| Modification Date | 2021-02-24T02:06:28 |
| Spectrometer Frequency | 100.63 |
| Spectral Width | 25000.0 |
| Lowest Frequency | -1433.4 |
| Nucleus | 13C |
| Acquired Size | 59522 |
| Spectral Size | 131072 |
| Digital Resolution | 0.19 |

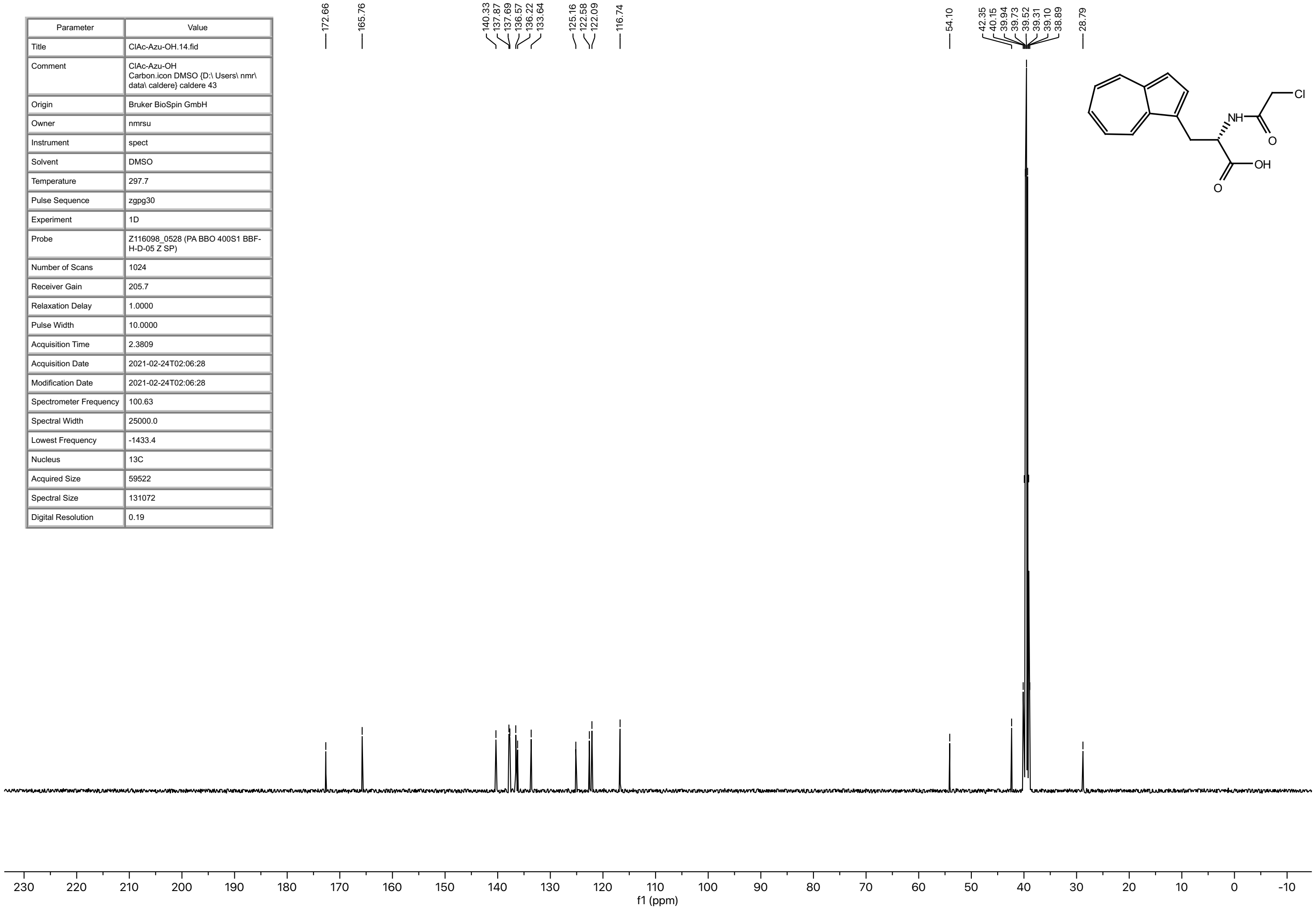

# *N*-Chloroacetyl 3-(azulen-1'-yl) alanine cyanomethyl ester (ClAc-AzAla-CME)

| Parameter | Value |
|---|---|
| Title | EC_YW_ClAc-Azu_CME.<br>100.fid |
| Comment | ClAc-Azu_CME<br>Proton.icon DMSO {D:\ Users\ nmr\ data\ caldere} caldere 60 |
| Origin | Bruker BioSpin GmbH |
| Owner | nmrsu |
| Instrument | spect |
| Solvent | DMSO |
| Temperature | 297.7 |
| Pulse Sequence | zg30 |
| Experiment | 1D |
| Probe | Z116098_0528 (PA BBO 400S1 BBF-H-D-05 Z SP) |
| Number of Scans | 128 |
| Receiver Gain | 91.2 |
| Relaxation Delay | 2.0000 |
| Pulse Width | 10.0000 |
| Acquisition Time | 2.0447 |
| Acquisition Date | 2021-03-13T04:35:24 |
| Modification Date | 2021-03-13T04:35:24 |
| Spectrometer Frequency | 400.15 |
| Spectral Width | 12019.2 |
| Lowest Frequency | -3541.7 |
| Nucleus | 1H |
| Acquired Size | 24576 |
| Spectral Size | 131072 |
| Digital Resolution | 0.09 |

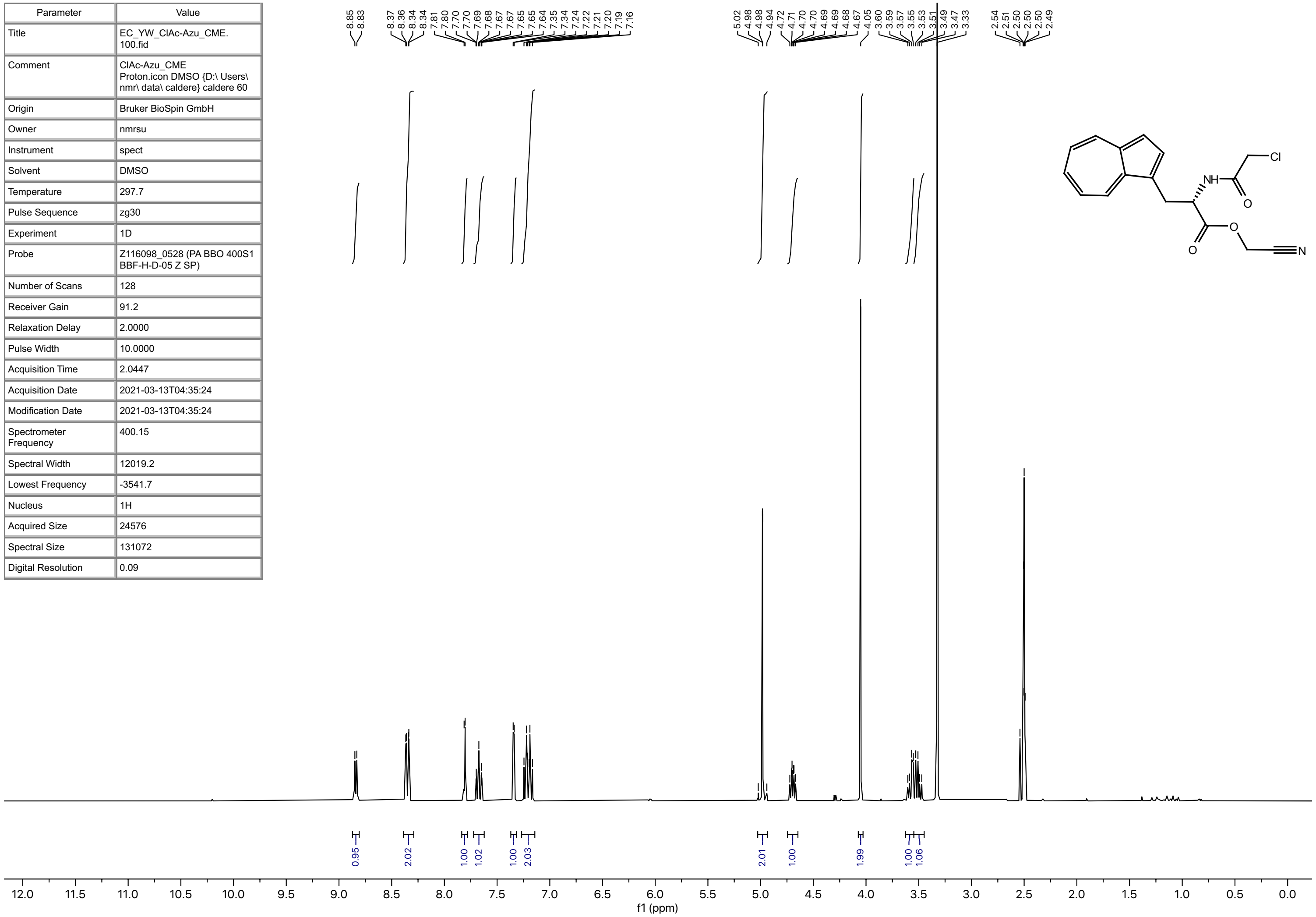

# *N*-Chloroacetyl 3-(azulen-1'-yl) alanine cyanomethyl ester (ClAc-AzAla-CME)

| Parameter | Value |
|---|---|
| Title | EC_YW_ClAc-Azu_CME.101.fid |
| Comment | ClAc-Azu_CME Carbon.icon DMSO {D:\ Users\ nmr\ data\ caldere} caldere 60 |
| Origin | Bruker BioSpin GmbH |
| Owner | nmrsu |
| Instrument | spect |
| Solvent | DMSO |
| Temperature | 297.7 |
| Pulse Sequence | zgpg30 |
| Experiment | 1D |
| Probe | Z116098_0528 (PA BBO 400S1 BBF-H-D-05 Z SP) |
| Number of Scans | 1024 |
| Receiver Gain | 205.7 |
| Relaxation Delay | 1.0000 |
| Pulse Width | 10.0000 |
| Acquisition Time | 2.3809 |
| Acquisition Date | 2021-03-13T05:35:36 |
| Modification Date | 2021-03-13T05:35:36 |
| Spectrometer Frequency | 100.63 |
| Spectral Width | 25000.0 |
| Lowest Frequency | -1481.3 |
| Nucleus | 13C |
| Acquired Size | 59522 |
| Spectral Size | 131072 |
| Digital Resolution | 0.19 |

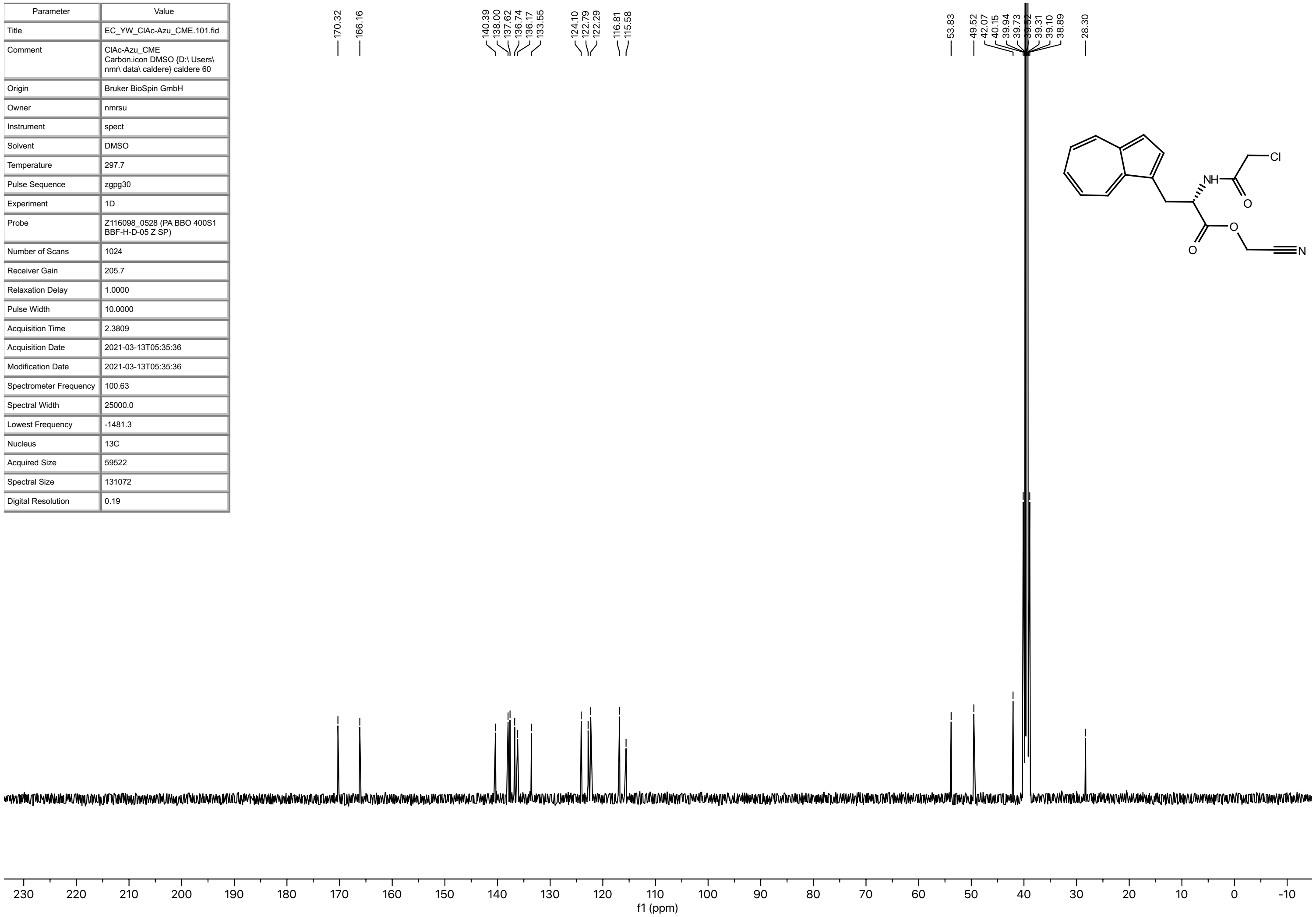

## *N*-Chloroacetyl 4-cyanotryptophan (ClAc-4CNW)

**Openlynx Report - JAMES** Page 1

| | | |
|---|---|---|
| Sample: 1 | Vial:2:31 | LNB Ref::JR-4 AcCl fraction 14 RP column |
| File:JR-4 AcCl fraction 14 RP column | Date:02-Dec-2019 | Time:16:46:53 |
| Submitter:JAMES | Method:C:\MassLynx\Acid_Col1_97-5_2min_1mL.olp | |
| Comment::JR-4 AcCl fraction 14 RP column | | |

Printed: Mon Dec 02 16:51:52 2019

**Sample Report:**

**Sample 1 Vial 2:31 ID JR-4 AcCl fraction 14 RP column File JR-4 AcCl fraction 14 RP column Date 02-Dec-2019 Time 16:46:53 Descriptic**

3: UV Detector: TAC: Wavelength Range: (210 - 400) Smooth (Mn, 2x3) 1.469e+2
Range: 1.482e+2

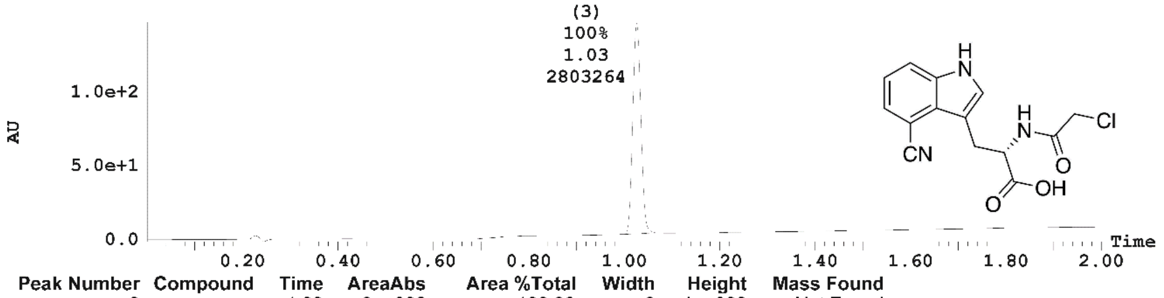


| Peak Number | Compound | Time | AreaAbs | Area %Total | Width | Height | Mass Found |
|---|---|---|---|---|---|---|---|
| 3 | | 1.03 | 3e+006 | 100.00 | 0 | 1e+008 | Not Found |

1: MS ES+ :TIC Smooth (Mn, 1x1) 1.7e+007

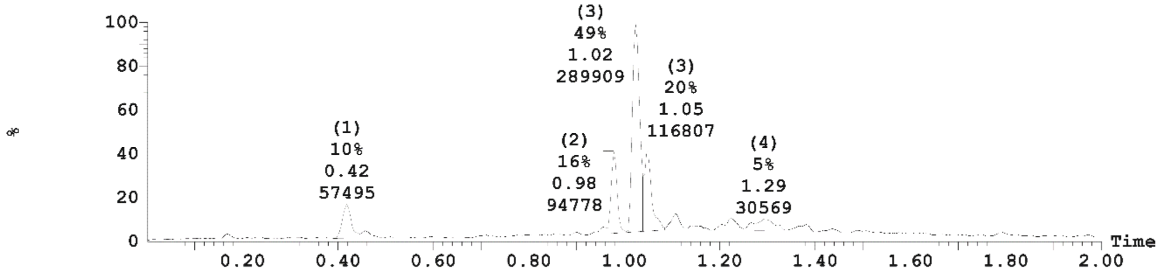


2: MS ES- :TIC Smooth (Mn, 1x1) 4.6e+006

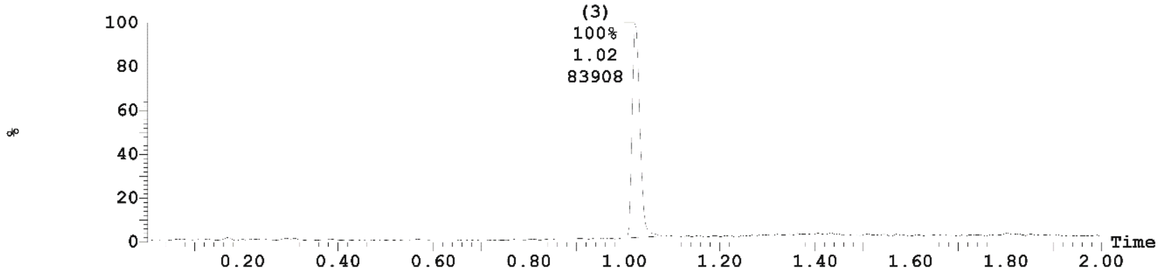

# *N*-Chloroacetyl 4-cyanotryptophan (ClAc-4CNW)

**Openlynx Report - JAMES** Page 2

| | | |
|---|---|---|
| Sample: 1 | Vial:2:31 | LNB Ref::JR-4 AcCl fraction 14 RP column |
| File:JR-4 AcCl fraction 14 RP column | Date:02-Dec-2019 | Time:16:46:53 |
| Submitter:JAMES | Method:C:\MassLynx\Acid_Col1_97-5_2min_1mL.olp | |
| Comment::JR-4 AcCl fraction 14 RP column | | |

Printed: Mon Dec 02 16:51:52 2019

**Sample Report (continued):**

| Peak ID | Compound | Time | Mass Found |
|---|---|---|---|
| 3 | | 1.02 | Not Found |

3:(Time: 1.03) Combine (256:282-(217:229+309:321)) 1:MS ES+ 1.1e+006

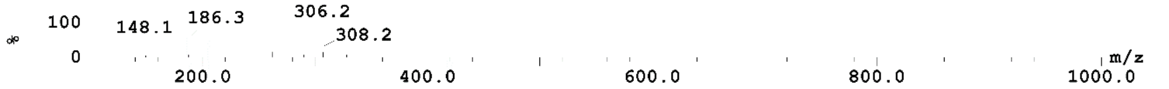


| Peak ID | Compound | Time | Mass Found |
|---|---|---|---|
| 3 | | 1.02 | Not Found |

3:(Time: 1.03) Combine (255:281-(216:228+308:320)) 2:MS ES- 2.2e+005

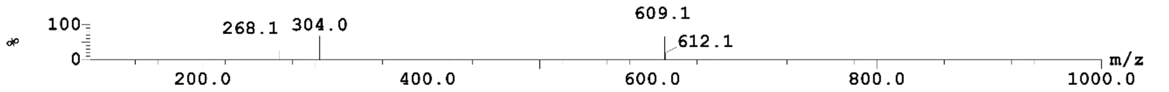

# *N*-Chloroacetyl 4-cyanotryptophan cyanomethyl ester (ClAc-4CNW-CME)

**Openlynx Report - ECALDER** Page 1

| | | |
|---|---|---|
| Sample: 1 | Vial:1:40 | LNB Ref::EC_YW_AcCl-4CNW-CME |
| File:EC_YW_AcCl-4CNW-CME | Date:09-Mar-2021 | Time:13:37:17 |
| Submitter:ECALDER | Method:C:\MassLynx\Acid_Col1_97-5_4min_0pt5mL.olp | |
| Comment:: | | |

Printed: Tue Mar 09 13:43:25 2021

**Sample Report:**

**Sample 1 Vial 1:40 ID EC_YW_AcCl-4CNW-CME File EC_YW_AcCl-4CNW-CME Date 09-Mar-2021 Time 13:37:17 Description**

3: UV Detector: TAC: Wavelength Range: (210 - 400) 3.984e+2
Range: 3.984e+2

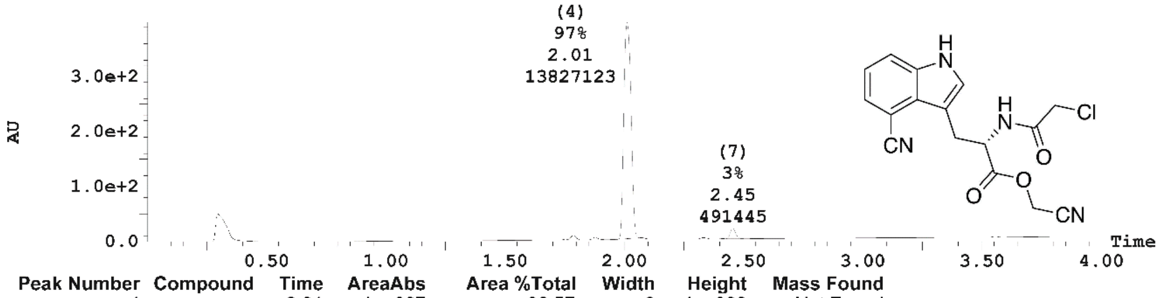


| Peak Number | Compound | Time | AreaAbs | Area %Total | Width | Height | Mass Found |
|---|---|---|---|---|---|---|---|
| 4 | | 2.01 | 1e+007 | 96.57 | 0 | 4e+008 | Not Found |
| 7 | | 2.45 | 5e+005 | 3.43 | 0 | 2e+007 | Not Found |

1: MS ES+ :TIC 1.9e+007

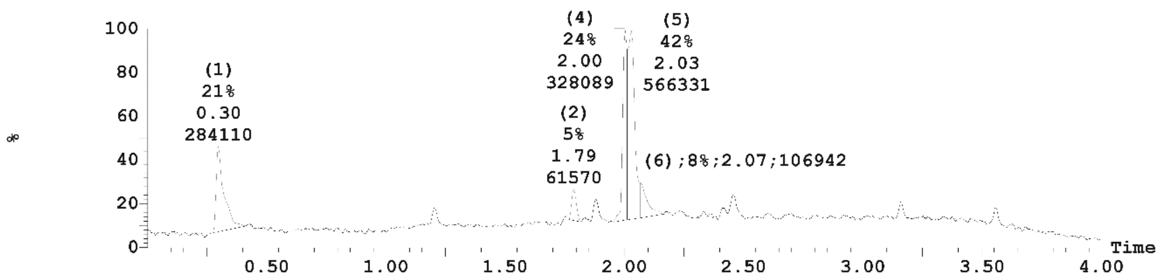


2: MS ES- :TIC 6.2e+006

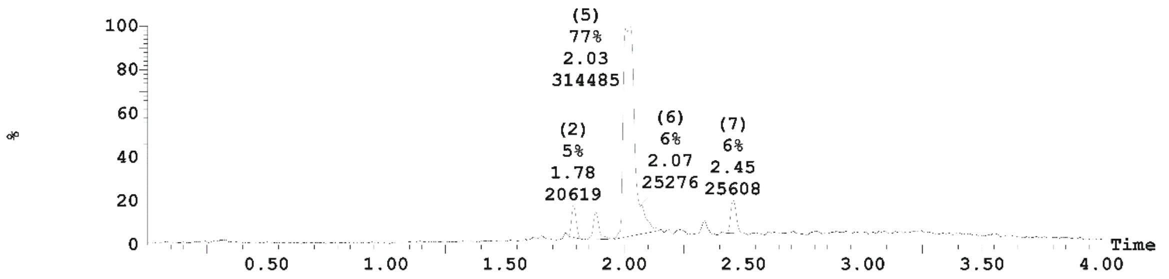

## *N*-Chloroacetyl 4-cyanotryptophan cyanomethyl ester (ClAc-4CNW-CME)



Sample: 1 Vial:1:40 LNB Ref::EC_YW_AcCl-4CNW-CME
File:EC_YW_AcCl-4CNW-CME Date:09-Mar-2021 Time:13:37:17
Submitter:ECALDER Method:C:\MassLynx\Acid_Col1_97-5_4min_0pt5mL.olp
Comment::

Printed: Tue Mar 09 13:43:25 2021

**Sample Report (continued):**

| Peak ID | Compound | Time | Mass Found |
|---|---|---|---|
| 4 | | 2.00 | Not Found |

4:(Time: 2.01) Combine (437:459-(403:414+482:492)) 1:MS ES+ 3.4e+006

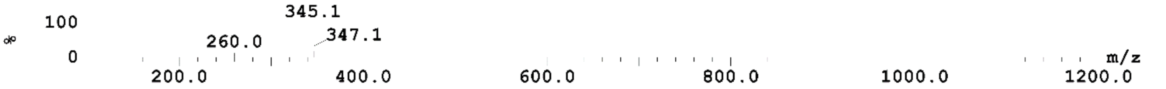


| Peak ID | Compound | Time | Mass Found |
|---|---|---|---|
| 7 | | 2.45 | Not Found |

7:(Time: 2.45) Combine (535:558-(502:512+581:591)) 1:MS ES+ 1.4e+005

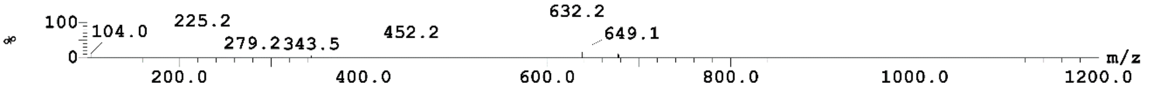


| Peak ID | Compound | Time | Mass Found |
|---|---|---|---|
| 4 | | 2.00 | Not Found |

4:(Time: 2.01) Combine (436:459-(403:413+482:492)) 2:MS ES- 1.7e+006

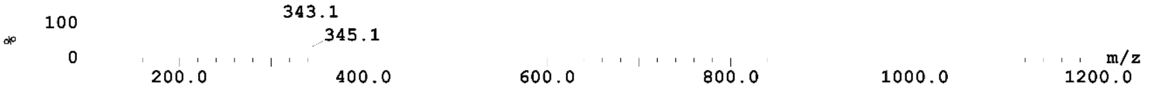


| Peak ID | Compound | Time | Mass Found |
|---|---|---|---|
| 7 | | 2.45 | Not Found |

7:(Time: 2.45) Combine (535:557-(502:512+580:591)) 2:MS ES- 5.0e+004

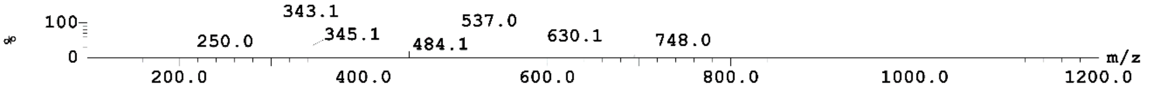

# *N*-Chloroacetyl 3-(azulen-1'-yl) alanine (ClAc-AzAla)

**Openlynx Report - MWu** Page 1

| | | |
|---|---|---|
| Sample: 1 | Vial:2:25 | LNB Ref::yw122-P1 |
| File:yw122-P1 | Date:29-Sep-2020 | Time:16:47:59 |
| Submitter:MWu | Method:C:\MassLynx\Acid_Col1_97-5_4min_0pt5mL.olp | |
| Comment:: | | |

Printed: Tue Sep 29 16:54:30 2020

**Sample Report:**

**Sample 1 Vial 2:25 ID yw122-P1 File yw122-P1 Date 29-Sep-2020 Time 16:47:59 Description**

3: UV Detector: TAC: Wavelength Range: (210 - 400) Smooth (Mn, 2x3) 2.367e+1
Range: 2.938e+1

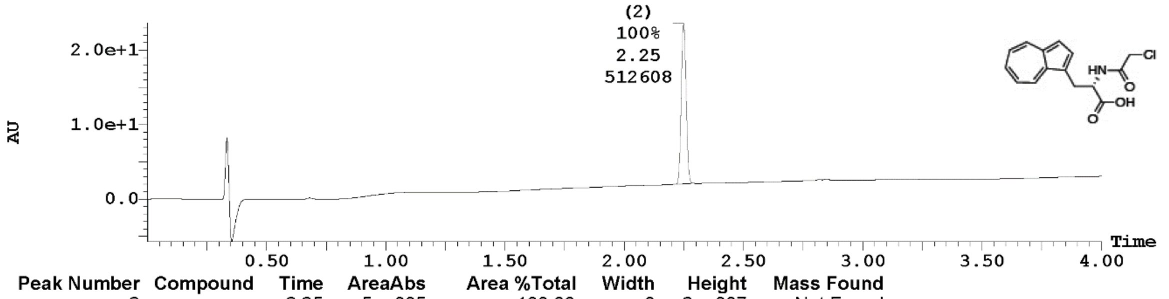


| Peak Number | Compound | Time | AreaAbs | Area %Total | Width | Height | Mass Found |
|---|---|---|---|---|---|---|---|
| 2 | | 2.25 | 5e+005 | 100.00 | 0 | 2e+007 | Not Found |

1: MS ES+ :TIC Smooth (Mn, 1x1) 5.5e+006

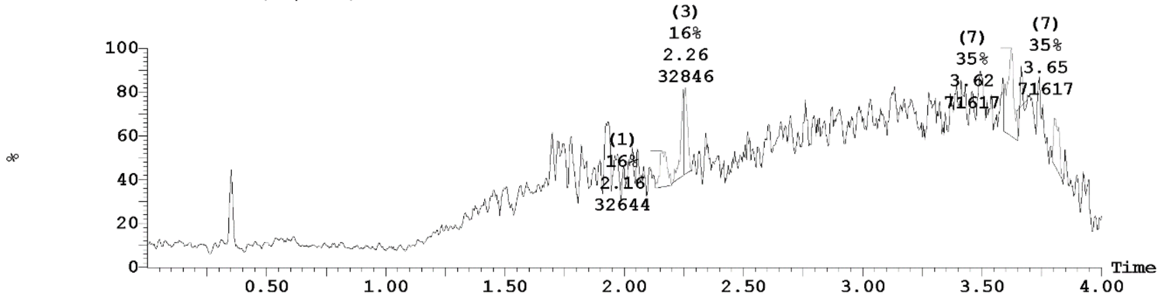


2: MS ES- :TIC Smooth (Mn, 1x1) 5.7e+005

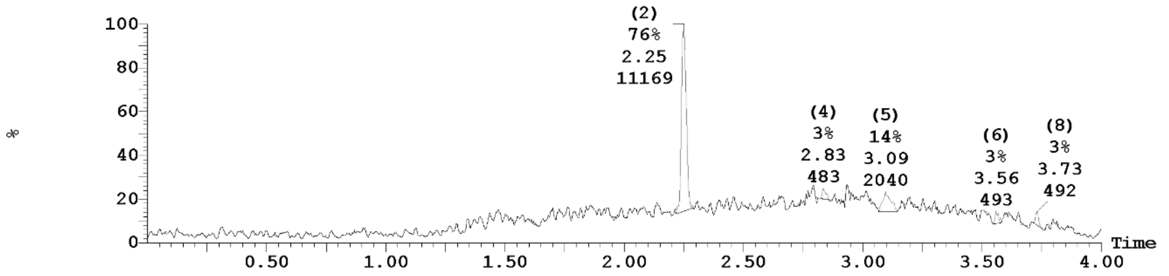

## *N*-Chloroacetyl 3-(azulen-1'-yl) alanine (ClAc-AzAla)

**Openlynx Report - MWu** Page 2

| | | |
|---|---|---|
| Sample: 1 | Vial:2:25 | LNB Ref::yw122-P1 |
| File:yw122-P1 | Date:29-Sep-2020 | Time:16:47:59 |
| Submitter:MWu | Method:C:\MassLynx\Acid_Col1_97-5_4min_0pt5mL.olp | |
| Comment:: | | |

Printed: Tue Sep 29 16:54:30 2020

**Sample Report (continued):**

| Peak ID | Compound | Time | Mass Found |
|---|---|---|---|
| 2 | | 2.25 | Not Found |

2:(Time: 2.25) Combine (489:511-(456:466+535:545)) 1:MS ES+ 2.7e+005

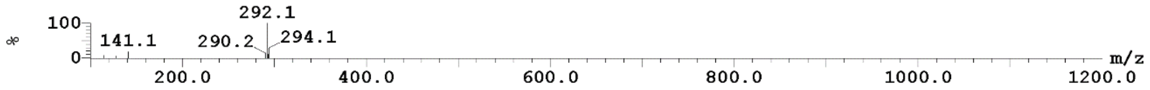


| Peak ID | Compound | Time | Mass Found |
|---|---|---|---|
| 2 | | 2.25 | Not Found |

2:(Time: 2.25) Combine (489:511-(455:466+534:544)) 2:MS ES- 4.5e+004

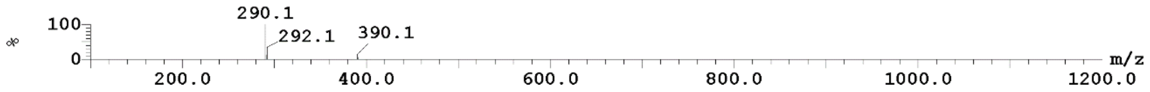

# *N*-Chloroacetyl 3-(azulen-1'-yl) alanine cyanomethyl ester(ClAc-AzAla-CME)

**Openlynx Report - MWu** Page 1

| | | |
|---|---|---|
| Sample: 1 | Vial:2:48 | LNB Ref::yw123-P1 |
| File:yw123-P1 | Date:30-Sep-2020 | Time:10:47:35 |
| Submitter:MWu | Method:C:\MassLynx\Acid_Col1_97-5_4min_0pt5mL.olp | |
| Comment:: | | |

Printed: Wed Sep 30 10:54:07 2020

**Sample Report:**

**Sample 1 Vial 2:48 ID yw123-P1 File yw123-P1 Date 30-Sep-2020 Time 10:47:35 Description**

3: UV Detector: TAC: Wavelength Range: (210 - 400) Smooth (Mn, 2x3) 1.561e+1
Range: 2.452e+1

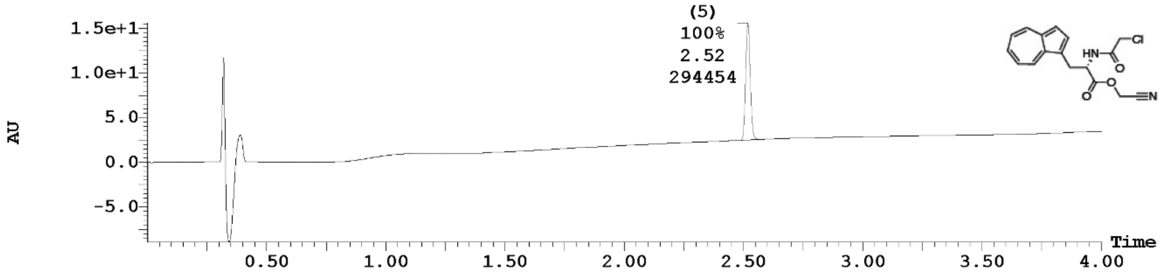


| Peak Number | Compound | Time | AreaAbs | Area %Total | Width | Height | Mass Found |
|---|---|---|---|---|---|---|---|
| 5 | | 2.52 | 3e+005 | 100.00 | 0 | 1e+007 | Not Found |

1: MS ES+ :TIC Smooth (Mn, 1x1) 5.0e+006

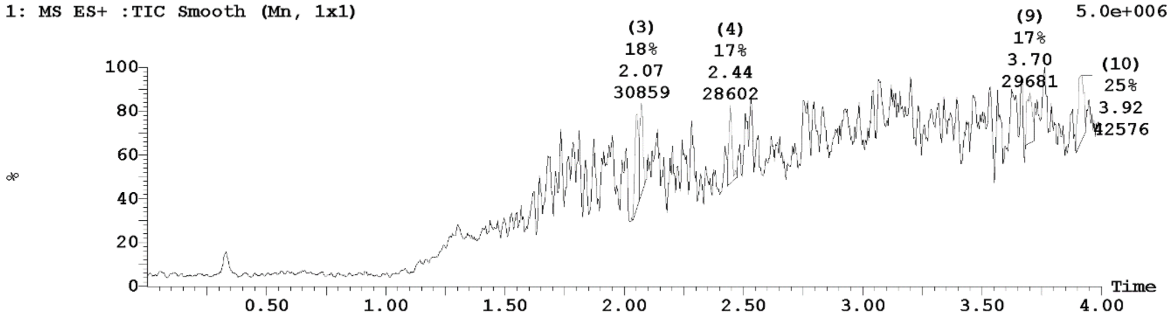


2: MS ES- :TIC Smooth (Mn, 1x1) 1.7e+005

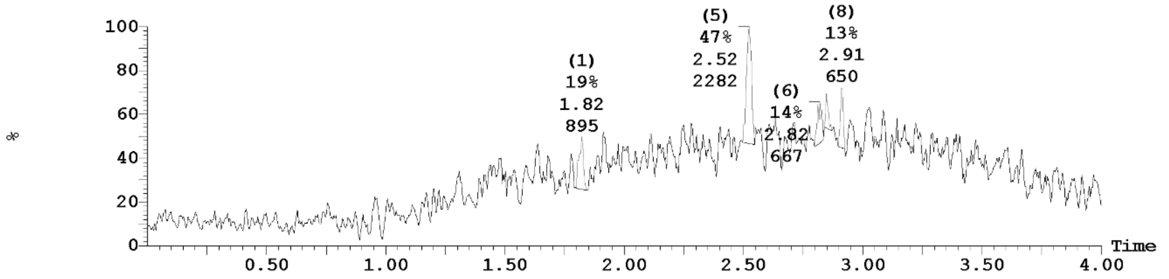

## *N*-Chloroacetyl 3-(azulen-1'-yl) alanine cyanomethyl ester(ClAc-AzAla-CME)

**Openlynx Report - MWu** Page 2

| | | |
|---|---|---|
| Sample: 1 | Vial:2:48 | LNB Ref::yw123-P1 |
| File:yw123-P1 | Date:30-Sep-2020 | Time:10:47:35 |
| Submitter:MWu | Method:C:\MassLynx\Acid_Col1_97-5_4min_0pt5mL.olp | |
| Comment:: | | |

Printed: Wed Sep 30 10:54:07 2020

**Sample Report (continued):**

| Peak ID | Compound | Time | Mass Found |
|---|---|---|---|
| 5 | | 2.52 | Not Found |

5:(Time: 2.52) Combine (549:572-(516:526+595:605)) 1:MS ES+ 1.6e+005

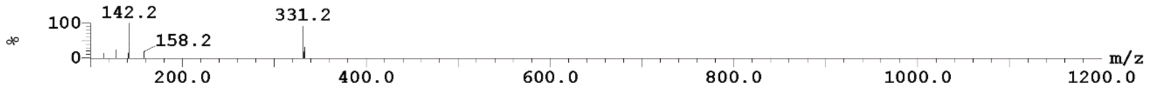


| Peak ID | Compound | Time | Mass Found |
|---|---|---|---|
| 5 | | 2.52 | Not Found |

5:(Time: 2.52) Combine (549:571-(515:526+594:604)) 2:MS ES- 4.1e+003

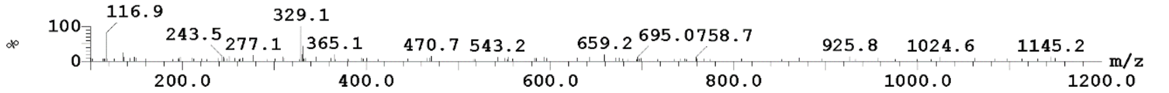

Supplement: Supplementary file 1 — Supporting Information [file CMDC-16-3185-s001.pdf]
